# *In vivo* Efficacy and Pharmacokinetics of Optimized Apidaecin Analogs

**DOI:** 10.3389/fchem.2017.00015

**Published:** 2017-03-20

**Authors:** Rico Schmidt, Daniel Knappe, Elisabeth Wende, Eszter Ostorházi, Ralf Hoffmann

**Affiliations:** ^1^Faculty of Chemistry and Mineralogy, Institute of Bioanalytical Chemistry, Universität LeipzigLeipzig, Germany; ^2^Center for Biotechnology and Biomedicine, Universität LeipzigLeipzig, Germany; ^3^Institute of Medical Microbiology, Semmelweis UniversityBudapest, Hungary

**Keywords:** Api88, Api137, *E. coli* ATCC 25922, intraperitoneal infection, intraperitoneal lavage, mass spectrometry, multiple reaction monitoring, organ homogenates

## Abstract

Proline-rich antimicrobial peptides (PrAMPs) represent promising alternative therapeutic options for the treatment of multidrug-resistant bacterial infections. PrAMPs are predominantly active against Gram-negative bacteria by inhibiting protein expression via at least two different modes of action, i.e., blocking the ribosomal exit tunnel of 70S ribosomes (oncocin-type binding) or inhibiting the assembly of the 50S ribosomal subunit (apidaecin-type binding). The *in vivo* efficacy and favorable biodistribution of oncocins confirmed the therapeutic potential of short PrAMPs for the first time, whereas the *in vivo* evaluation of apidaecins is still limited despite the promising efficacy of apidaecin-analog Api88 in an intraperitoneal murine infection model. Here, the *in vivo* efficacy of apidaecin-analog Api137 was studied, which rescued all NMRI mice from a lethal intraperitoneal infection with *E. coli* ATCC 25922 when administered three times intraperitoneal at doses of 0.6 mg/kg starting 1 h after infection. When Api88 and Api137 were administered intravenous or intraperitoneal at doses of 5 and 20 mg/kg, their plasma levels were similarly low (<3 μg/mL) and four-fold lower than for oncocin-analog Onc72. This contradicted earlier expectation based on the very low serum stability of Api88 with a half-life time of only ~5 min compared to ~6 and ~3 h for Api137 and Onc72, respectively. Pharmacokinetic data relying on a sensitive mass spectrometry method utilizing multiple reaction monitoring and isotope-labeled peptides revealed that Api88 and Api137 were present in blood, urine, and kidney, and liver homogenates at similar levels accompanied by the same major metabolites comprising residues 1–16 and 1–17. The pretended discrepancy was solved, when all peptides were incubated in peritoneal lavage. Api137 was rapidly degraded at the C-terminus, while Api88 was rather stable despite releasing the same degradation products. Onc72 was very stable explaining its higher plasma levels compared to Api88 and Api137 after intraperitoneal administration illuminating its good *in vivo* efficacy. The data indicate that the degradation of therapeutic peptides should be studied in serum and further body fluids. Moreover, the high efficacy in murine infection models and the fast clearance of Api88 and Api137 within ~60 min after intravenous and ~90 min after intraperitoneal injections indicate that their *in vivo* efficacy relates to the maximal peptide concentration achieved in blood.

## Introduction

Antimicrobial peptides (AMPs) are considered as promising alternatives to common antibiotics that threateningly lose their therapeutic potentials against multidrug-resistant pathogens (Fox, 2013; Wang et al., 2015). In contrast to approved therapeutic peptide hormones, antimicrobial peptides have to be administered at much higher doses, which may amplify general drawbacks of peptide therapeutics. For example, the diabetes peptide drugs exenatide and pramlintide are given at daily doses of 20 μg (Byetta®; ~0.5 μg/kg) and 120 μg (Symlin®; ~1.5 μg/kg; Heine et al., 2005; Hay et al., 2015), while antimicrobial peptides are usually administered at 50- to 100-fold higher doses (>5 mg/kg) in order to achieve high *in vivo* efficacies (Benincasa et al., 2010; Szabo et al., 2010; Brunetti et al., 2016). This is also true for proline-rich AMPs (PrAMPs), such as insect-derived oncocin and apidaecin analogs that showed protective effects in murine intraperitoneal and intramuscular infections with *Escherichia coli* ATCC 25922 and antibiotic-susceptible and -resistant *Klebsiella pneumoniae* strains (Czihal et al., 2012; Knappe et al., 2012, 2015; Ostorhazi et al., 2014; Schmidt et al., 2016). Mechanistically, cationic AMPs first electrostatically interact with the negatively charged surface of bacteria and—depending on the secondary structure—act on the membrane or reach the periplasmic space possibly by spontaneous translocation (Hancock, 1997; Brogden, 2005; Scocchi et al., 2016). PrAMPs exhibit a therapeutically favorable intracellular mode of action after translocating into the bacterial cytoplasm by utilizing different transporter proteins/complexes, such as SbmA, YjiL/MdtM, and YgdD (Mattiuzzo et al., 2007; Runti et al., 2013; Krizsan et al., 2015a; Paulsen et al., 2016). Internalized PrAMPs bind to chaperone DnaK and 70S ribosomes. While DnaK is most likely a binding partner with possible transport functions (Otvos et al., 2000; Knappe et al., 2011b, 2016a; Czihal et al., 2012; Zahn et al., 2013), the 70S ribosome appears to be the major bacterial target. For example, oncocin analogs Onc72 and Onc112 bind with K_d_ values of 450 and 90 nmol/L, respectively, in a fluorescence polarization assay to the exit tunnel, as recently revealed by X-ray crystallography (Krizsan et al., 2014; Roy et al., 2015; Seefeldt et al., 2015). Apidaecin-derived analogs Api88 and Api137 possess the same sequence, i.e., gu-ONNRPVYIPRPRPPHPRL (O: ornithine, gu: *N*,*N*,*N*′,*N*′-tetramethylguanidino), and differ only by the C-terminal amide and acid groups, respectively. They bind to 70S ribosomes with considerably lower affinities (K_d_ = 1.2 and 0.56 μmol/L, respectively). Surprisingly, only oncocins significantly inhibit cell free protein expression with IC_50_ values of ~0.2 μmol/L, whereas both apidaecins and oncocins inhibit protein translation in *E. coli* equally efficient with IC_50_ values of ~2 μmol/L. This different *in vitro* behavior appears to be related to different inhibition mechanisms. Api137 disturbs the assembly of the 50S ribosomal subunit in *E. coli* leading to a protein complex size of around 42S (Krizsan et al., 2015b). This unique mode of action leads to high antimicrobial activities against *E. coli* ATCC 25922 (MIC = 4 μg/mL) and other Gram-negative bacteria. Both Api88 and Api137 possess *in vitro* very similar antibacterial activities, but Api88 is much faster degraded in mouse serum *in vitro* with a low half-life time of only 5 min compared to 6 h for Api137 (Berthold et al., 2013). Api88 is degraded C-terminally to virtually inactive Api1-17 and Api1-16. Despite its low serum stability *in vitro*, Api88 was highly efficient in a lethal NMRI mouse model of intraperitoneal sepsis providing 100% survival rates at doses of 1.25 mg/kg (Czihal et al., 2012).

In the present study, we investigated the efficacy of Api137 in the same NMRI mouse infection model, which indeed turned out to be even better than for Api88. Focusing on the pharmacokinetics, Api88 and Api137 were quantified by reversed-phase (RP-) HPLC coupled online to an electrospray ionization (ESI) mass spectrometer (MS) utilizing multiple reaction monitoring (MRM) relative to isotope-labeled peptide standards. Both peptides and their two major metabolites were studied in blood, urine, and homogenates of kidney, liver, and brain. Together with previous reports on the pharmacokinetics and *in vivo* efficacy of Onc72 and Onc112 (Knappe et al., 2012; Holfeld et al., 2015; Schmidt et al., 2016) the results presented here for Api88 and Api137 provide the first comprehensive representation of the therapeutic potential of insect-derived PrAMPs.

## Materials and methods

### Peptides

Apidaecin derivatives (Table S1) were synthesized on solid phase using the 9-fluorenylmethoxycarbonyl/*tert*-butyl (Fmoc/^t^Bu)-strategy and *in situ* activation with *N*,*N*′-diisopropylcarbodiimide in the presence of *N*-hydroxybenzotriazole as described previously (Czihal et al., 2012; Berthold et al., 2013). Isotope-labeled Fmoc-Pro-OH [97–99% ^13^C_5_, 97–99% ^15^N] (Euriso-Top GmbH, Saarbrücken, Germany) was coupled manually overnight using a 1.5-fold excess of the amino acid derivative. Peptides were cleaved with TFA containing a scavenger mixture (12.5% v/v; ethandithiole, m-cresol, water, and thioanisole, 1/2/2/2 v/v/v/v) and afterwards precipitated and washed three times with cold diethyl ether. Peptides were purified by RP-HPLC on a Jupiter C_18_-column (ID: 10 mm) using a linear aqueous acetonitrile gradient containing TFA (0.1% v/v) as ion pair reagent. Purities were judged by RP-HPLC and the masses were confirmed by MALDI- or ESI-MS.

### Mass spectrometry

A protocol to quantify Api88, Api137, and their major metabolites was established analog to that of oncocin derivatives, reported recently (Schmidt et al., 2016). Briefly, an Alliance® 2695 HPLC system was coupled online to an ESI-QqLIT-MS (4,000 QTRAP®) equipped with a TurboV™ ion-spray source and operated in positive ion MRM mode using the Analyst® 1.6 software. Separation was achieved on a Jupiter C_18_-column (internal diameter 1 mm, length 150 mm, particle size 5 μm, pore size 300 Å) at 55 °C using a linear 5-min gradient from 2.7 to 20.7% aqueous acetonitrile containing ammonium formate (26 mmol/L, pH 3.0). Further details about instrumentation, MRM optimization, and MS settings are provided as Supplementary Material (Tables S1–S4, Method M1).

Metabolites of Api88 and Api137 were identified on a nanoACQUITY UPLC® System coupled online to an ESI-LTQ Orbitrap XL™-MS (for details see Tables S2, S5). Peptides enriched by solid phase extraction were separated on a BEH C_18_ nanoACQUITY UPLC® column (100 μm internal diameter, 100 mm length, 1.7 μm particle size, 13 nm pore size, 30°C) using an aqueous acetonitrile gradient from 3 to 30% (within 18 min) and 30 to 95% acetonitrile (1 min) in the presence of formic acid (0.1% v/v) at a flow rate of 0.4 μL/min. Differences in the peptide profiles were identified by comparing the extracted ion chromatograms (XICs, *m/z* ± 0.01) of samples obtained from treated and untreated mice. MassLynx™ software was used to control the nanoACQUITY UPLC® and the Orbitrap MS and Xcalibur™ software to process MS data.

### Animals and housing

Female outbred NMRI mice (Janvier Labs, Saint Berthevin Cedex, France) were housed in an individually ventilated cage system (Ebeco, Castrop-Rauxel, Germany) under pathogen-free conditions with water and standard food (R/M-H, ssniff Spezialdiäten GmbH, Soest, Germany) given *ad libitum*. After acclimatization for at least one week, studies were performed according to the guidelines for the Care and Use of Laboratory Animals and the study was approved by the Animal Care and Usage Committee of the state agency Leipzig (Landesdirektion Leipzig, file numbers 24-9168.11/14/37 and 24-9168.11/17/43).

Female outbred CD-1 mice (Innovo, Gödöllő, Hungary) were acclimatized 1 week after arrival in a sterile plastic type 2 cage (Innovo Kft., Gödöllő) on softwood granules with free access to water and sterile pelleted rodent food (Szinbád Ltd., Gödöllő). All animals were cared for and experiments were performed in accordance with the recommendations of the Guidelines for the Care and Use of Laboratory Animals and the study was approved by the Animal Care Committees of Semmelweis University (permission No.: 001/2218-4/2012).

### Systemic septicemia infection mouse model

The acute toxicity of Api137 was studied by administering doses of 20, 40, 80, and 120 mg/kg intraperitoneally (i.p.) four times daily (0, 3, 7, and 24 h) into female NMRI mice (24–32 g, ~7 weeks old). For *in vivo* efficacy, female NMRI mice (high dose experiment: 24–27.1 g, 6 weeks; low dose experiment: 26.8–30.3 g, 7 weeks) were infected intraperitoneal with *E. coli* ATCC 25922, as described before (Czihal et al., 2012; Knappe et al., 2012). Briefly, each mouse was infected with 9 × 10^5^ bacteria in the presence of 2.5% (w/v) mucin (0.3 mL in total). Mucin restricts acute macrophage activation in the intraperitoneal space and promotes establishment of a reproducible infection (Frimodt-Møller et al., 1999). Mice were observed three times daily for their health status for a total of 5 days post-infection and weighed 1 day before and 1 and 5 days after infection. In accordance with the guidelines of the Animal Care and Usage Committee of the Landesdirektion Leipzig, moribund mice were euthanized.

Apidaecin Api137 (high dose experiment: 2.5 and 1.25 mg/kg body weight; low dose experiment: 0.6 and 0.3 mg/kg body weight) was administered intraperitoneal (0.3 mL) in seven mice per group three times post-infection (1, 4, and 8 h) as antibacterial therapy. Each experiment included seven mice receiving ciprofloxacin (40 mg/kg body weight) and four mice receiving only the vehicle (5% glucose in water, w/v) as positive and negative controls, respectively.

### Pharmacokinetics

Female NMRI mice (8–9 weeks, 25–35 g) were injected intraperitoneally (10 mL/kg) with peptides dissolved in sterile PBS (Gibco® Life Technologies™, Darmstadt, Germany) at concentrations of 0.5 or 2 g/L corresponding to doses of 5 or 20 mg/kg body weight, respectively. After 10, 20, 30, 60, or 90 min animals (*n* = 7) were euthanized by carbon dioxide inhalation and terminal bleeding via cardiac puncture. Lithium-heparin plasma (*n* = 7) was prepared and stored at −80°C. Immediately after the final bleed, mice were perfused through the left ventricle with saline (0.9%, 50 mL, ~3 min) to remove blood from all organs controlled by discoloration of the liver. Organs were removed aseptically and directly frozen in liquid nitrogen. Spontaneously released urine was collected and frozen immediately at −80°C.

Female CD-1 mice (20–23 g weight, 7 weeks) were injected intravenously (10 mL/kg) in the tail vein with peptides (0.5 g/L) dissolved in sterile saline (0.9%, w/v) corresponding to doses of 5 mg/kg body weight. Animals (*n* = 4 per time point and peptide) were euthanized 5, 10, 20, 40, and 60 min post-injection by carbon dioxide inhalation and exsanguination. For further details of sample preparation and recovery experiments see Supplementary Material (Methods M2, M3).

### Peptide stability in peritoneal lavage

Peritoneal lavage (1.5–2 mL) were collected from three euthanized female CD-1 mice (14 weeks, 32–33 g) after injecting sterile PBS intraperitoneal (4 mL). Peptides were incubated with aliquots (95 μL) of fresh lavage (75 μg/mL, 37°C, 750 rpm). After 0, 30, and 60 min trichloroacetic acid (25 μL, 15% w/v) was added to the samples to precipitate proteins, incubated on ice (10 min), and centrifuged (5 min, 12.400 × g). Supernatants were neutralized with sodium hydroxide (1 mol/L) and analyzed by RP-HPLC using a Poroshell 120 SB-C_18_-column (2.1 mm internal diameter, 100 mm length, 2.7 μm particle size, 12 nm pore size, Agilent Technologies Inc., Santa Clara, CA, USA) at 60°C and an aqueous acetonitrile gradient containing trifluoroacetic acid (0.1%, v/v). Absorbance was recorded at a wavelength of 214 nm.

## Results

### Intraperitoneal toxicity and therapy

When Api137 was administered intraperitoneally four times (0, 3, 7, and 24 h) at doses of 20, 40, and 80 mg/kg per injection, none of the seven animals per dose group showed any signs of adverse side effects. Higher doses of 120 mg/kg induced signs of discomfort in some animals and decreased mobility for up to 20 min after administration, but they recovered completely prior to the next treatment and all mice survived the observation period of 5 days.

**Figure 1:**
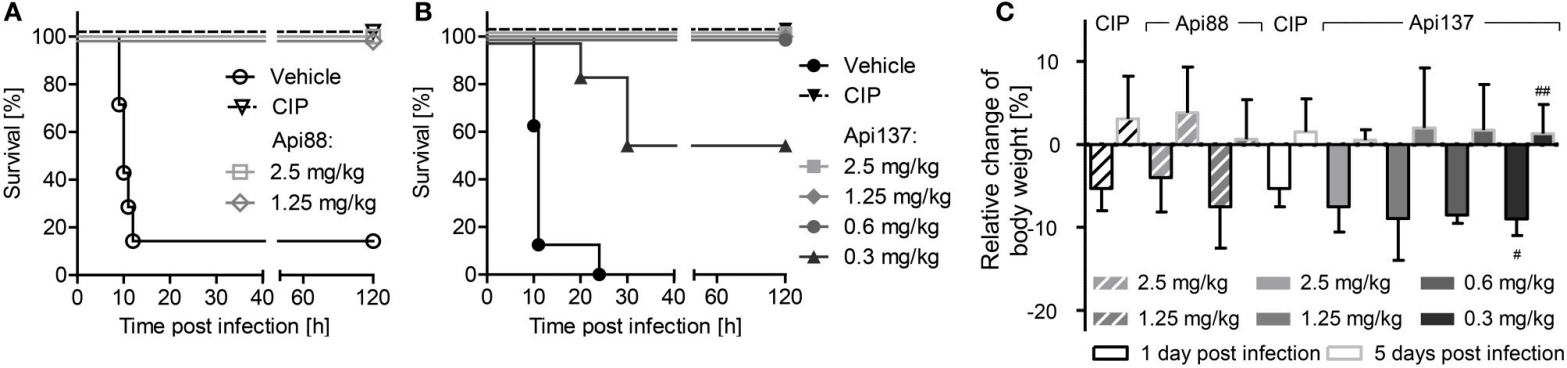
**Survival rates (A,B)** and relative change of body weights **(C)** of NMRI mice (*n* = 7) infected intraperitoneally with *E. coli* ATCC 25922 (9 × 10^5^ bacteria in the presence of mucin, 2.5% w/v, 300 μL in total). Api88 **(A)** and Api137 **(B)** were administered intraperitoneally (300 μL) at doses of 2.5 and 1.25 mg/kg and 2.5, 1.25, 0.6, and 0.3 mg/kg, respectively, three times post-infection (1, 4, and 8 h). Ciprofloxacin (CIP, 40 mg/kg body weight) and vehicle only (glucose in water, 5% w/v) were used as positive and negative control, respectively. Mice were controlled three times daily for their health status for 5 days post-infection and weighed 1 day before and 1 and 5 days after infection. Relative changes of body weights were calculated for Api88, Api137, and ciprofloxacin at 1 and 5 days post-infection (black and gray bordered bars, respectively). # and ## denote that 6 and 4 survivors were weighed, respectively.

As reported previously, Api88 administered intraperitoneally rescued all NMRI mice in a lethal *E. coli* ATCC 25922 infection model at doses of 2.5 and 1.25 mg/kg (Figure 1A; Czihal et al., 2012). Lower doses of Api88 were not tested, as the scoring of at least four animals in the low dose group (1.25 mg/kg) indicated that a further reduction of the dose would have led to very serious signs of infection and reduced weight (>20%) demanding euthanasia according to the guidelines of the ethical committee. Api137 appeared more efficient in this septicemia model due to the better scores obtained at the same dose and by rescuing all animals at doses of 0.6 mg/kg and higher and still four out of seven mice at a dose of 0.3 mg/kg, which represented approximately the ED_50_ (Figure 1B). The higher efficacy of Api137 had no detectable effect on body weights (Figure 1C), as the body weight losses of the highest dose groups of Api137 and Api88 (2.5 and 1.25 mg/kg) were comparable. The weight gain after 5 days post-infection lead to values slightly above the pre-infection values. Interestingly, animals treated with 2.5 mg/kg Api88 or Api137 showed similar weight losses as mice treated with ciprofloxacin at the recommended dose of 40 mg/kg within the respective experiment. The comparison of both peptides considering the corresponding ciprofloxacin controls indicates a slightly better outcome regarding the body weights for Api88, which may rely on the biological variance.

### Pharmacokinetics

A sensitive RP-HPLC-ESI-MS approach for targeted quantification of Api88, Api137, and their identical metabolites containing the N-terminal 16 (Api1-16) or 17 residues (Api1-17) was developed based on a protocol recently reported for oncocins (Schmidt et al., 2016). The MRM relied on triply protonated precursor ions and a neutral loss of two molecules of dimethylamine from the N-terminal *N*,*N*,*N*′,*N*′-tetramethylguanidine group, which was highly selective and sensitive providing limits of quantification (LOQ) from 19 to 59 ng/mL (~9 to ~25 nmol/L). Recovery rates were 35 ± 5–56 ± 16% after sample preparation with only weak matrix effects (−4.4 ± 0.2–13 ± 3%). Accuracies were 89 ± 6–116 ± 17% and intra- and interday precisions were 10% or better for all peptides and concentrations. Details of the method development and validation of the optimized method are provided as Supplementary Material (Tables S4, S6–S9, Figures S1–S3).

**Figure 2:**
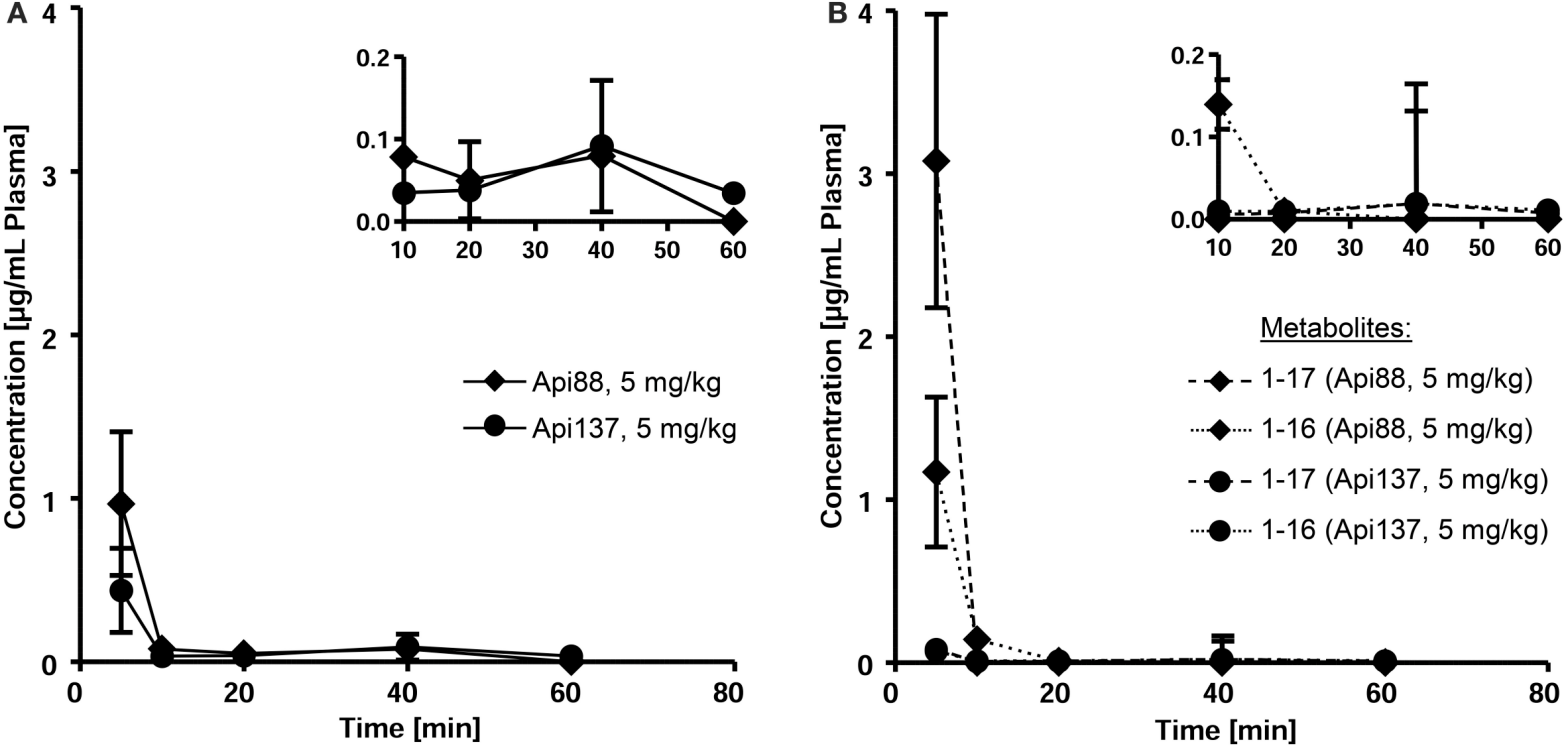
**Pharmacokinetic studies of Api88 and Api137 (A)** after intravenous administration at doses of 5 mg/kg BW and the corresponding metabolites Api1-16 and Api1-17 **(B)**. Peptides were quantified by RPC-ESI-QqLIT-MS for blood samples collected 5, 10, 20, 40, and 60 min after peptide administration (*n* = 5). Inserts display the same data points using zoomed concentration ranges.

Api88 administered intravenously at a dose of 5 mg/kg was detected at a plasma concentration of 1 μg/mL at the first time point after 5 min (Figure 2A). Unexpectedly, Api137, which has a 70 times longer half-life time in mouse serum, injected at the same dose was detected at a lower concentration of only 0.4 μg/mL in plasma (Figure 2A). The plasma levels of both peptides further decreased at similar kinetics to concentrations around the LOQ after 10 min. After 60 min Api137 was still present at concentrations around the LOQ, while Api88 was not detected.

**Figure 3:**
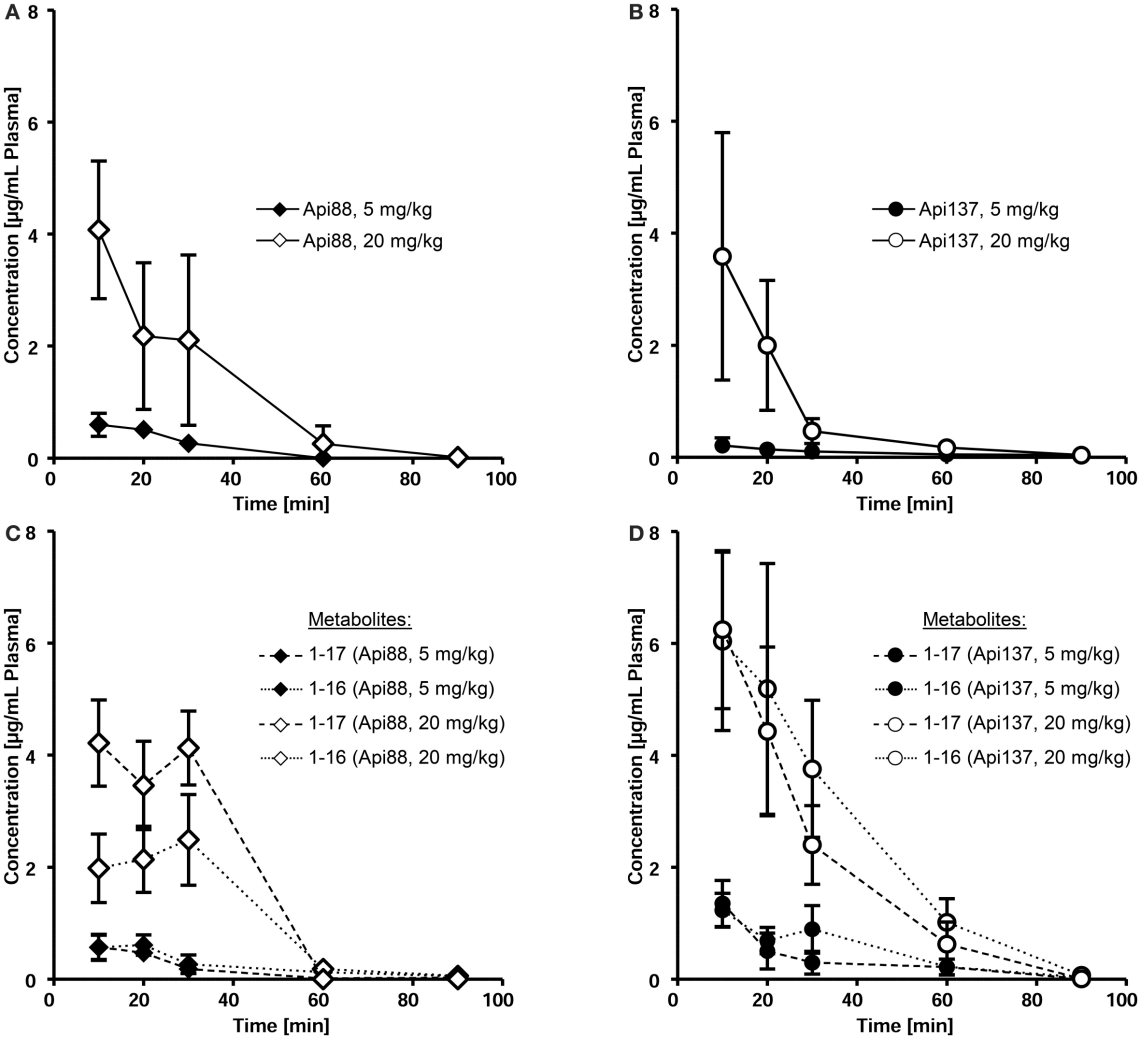
**Pharmacokinetics of Api88 (A)** and Api137 **(B)** and metabolites 1–17 and 1–16 **(C,D)** after intraperitoneal administration at doses of 5 mg/kg BW and 20 mg/kg BW. Peptides were quantified by RPC-ESI-QqLIT-MS/MS in plasma samples collected at 10, 20, 30, 60, and 90 min post-injection (*n* = 7).

Intraperitoneal applications were initially used in the infection models due to the presumed depot effect providing slower releases and thus longer circulation times in blood. Indeed, when Api88 and Api137 were injected intraperitoneal at doses of 5 mg/kg, slightly lower plasma levels of 0.6 and 0.2 mg/mL, respectively, were observed after 10 min that decreased even further during the next 20 min. Api137 was still detected after 1.5 h, whereas Api88 was undetectable (Figures 3A,B, Figures S4A,B, S5). The calculated initial concentrations (c_0_) were 0.2 and 1 μg/mL, respectively (Table 1), but the plasma levels decreased slower than for intravenous applications demonstrating a rather small depot effect. Again, Api88 was detected at the initial time points at higher plasma levels than Api137, but the elimination half-life time of Api137 (t_½_ = 33.8 min) was almost two-fold longer than for Api88 (t_½_ = 17.3 min), which could be explained by the higher serum stability of Api137.

**Table 1:** **Mean pharmacokinetic parameters of Api88 and Api137 in mice, after intraperitoneal administration, determined with PKSolver using the concentrations for the terminal elimination phase determined by the software**.

**Peptide**	**Api88**		**Api137**	
Dosage [mg/kg BW]	5	20	5	20
Injected peptide amount [μg]	137	618	145	602
c_max_[μg/mL]	0.6	4.1	0.2	3.6
c_0_[μg/mL]	1.0	6.2	0.22	7.8
t_1/2_[min]	17.3	11.1	33.8	16.9
k [min^−^^1^]	0.040	0.062	0.020	0.041
Volume of distribution [mL]	137	100	659	77
AUC_0 → ∞_ [μg^*^min^*^mL^−^^1^]	21.3	114.2	9.6	72.0
AUMC_0 → ∞_ [μg^*^min^2*^mL^−^^1^]	650	2730	1512	524
Clearance [mL/min]	6.4	5.4	15.1	8.4
Mean residence time [min]	30.5	23.9	54.9	21.0

*c_max_, c_0_, t_1/2_, k, AUC, and AUMC denote maximum concentration, initial concentration at time point 0 min, elimination half life time, elimination constant, area under the concentration time curve, and area under the moment curve, respectively*.

Four-fold higher doses (20 mg/kg) provided maximal plasma levels of 4.1 (Api88) and 3.6 μg/mL (Api137) after 10 min, which was 7- and 17-fold higher than observed for the low dose (Figures 3A,B). Surprisingly, the c_0_ values were similar for both peptides (6.2 and 7.8 μg/mL, respectively), while the elimination half-life times decreased to 11 and 17 min, respectively (Table 1). However, the data points indicated an almost stable plasma level for Api88 over 30 min, whereas Api137 was cleared faster resulting in a lower plasma level at 30 min. Although difficult to interpret, a comparison of the pharmacokinetic parameters clearance and mean residence time of the high and low dose groups seemed to be similar for Api88 and different for Api137.

### Metabolites in blood

Metabolites Api1-16 and Api1-17, which were identified as major degradation products of both Api88 and Api137 in serum stability assays (Berthold et al., 2013), were quantified besides the full-length peptides by RP-HPLC-ESI-QqLIT-MS using the optimized MRM method. As expected from serum stability assays, metabolites Api1-17 and Api1-16 appeared at higher concentrations (1.2 and 2.9 μg/mL after 10 min, respectively) for Api88 than for Api137 (<0.1 μg/mL) after intravenous administration at a dose of 5 mg/kg (Figure 2B). Although, the low concentrations of both Api137 metabolites are in full agreement with the higher *in vitro* stability of Api137, the total quantities of intact peptide and metabolites was significantly lower than for Api88.

Intraperitoneal administration yielded Api88, Api1-16, and Api1-17 at similar plasma levels that remained relatively stable for the first 30 min (Figure 3C, Figure S4C) indicating again a depot effect in the peritoneum. In contrast, both metabolites of Api137 were detected at an equal level that was around six-fold higher than for the intact peptide after 10 min administered at a dose of 5 mg/kg (Figure 3D, Figure S4D). For the high dose group, the ratio was initially around 2. Interestingly, the plasma levels of the metabolites remained above the levels of Api137 for the first hour and the curves showed a more exponential shape than for Api137 indicative of a slower clearance rate. The two administration routes resulted in clearly different metabolite profiles for both apidaecins. The higher serum stability of Api137 was not reflected *in vivo* when it was injected in the peritoneum. Altogether, the plasma levels of both Api88 and Api137 were lower than assumed from previous studies on oncocins, even when considering the major metabolites Api1-16 and Api1-17. Thus, the plasma samples obtained 10, 30, and 60 min after intraperitoneal injections were screened for further degradation products of both peptides using nanoRP-UPLC-ESI-LTQ-Orbitrap XL^TM^-MS (Figure S6). For Api88, five further metabolites with peak areas of 1 to 3% relative to the full length peptide were identified (Figure S6A), i.e., Api2-16, Api7-18, Api3-18, Api7-12, and Api1-6 in the order of decreasing peak areas. Surprisingly, besides Api1-16 and Api1-17, only three metabolites were detected for Api137 at low intensities (peak areas <1% relative to Api137), i.e., Api7-12, Api2-16, and Api2-18 in the order of decreasing peak areas (Figure S6B). However, numerous metabolites with very low intensities were observed indicating subsequent degradations, but their contents did not explain the unexpectedly fast clearance rates.

### Organ distribution

**Figure 4:**
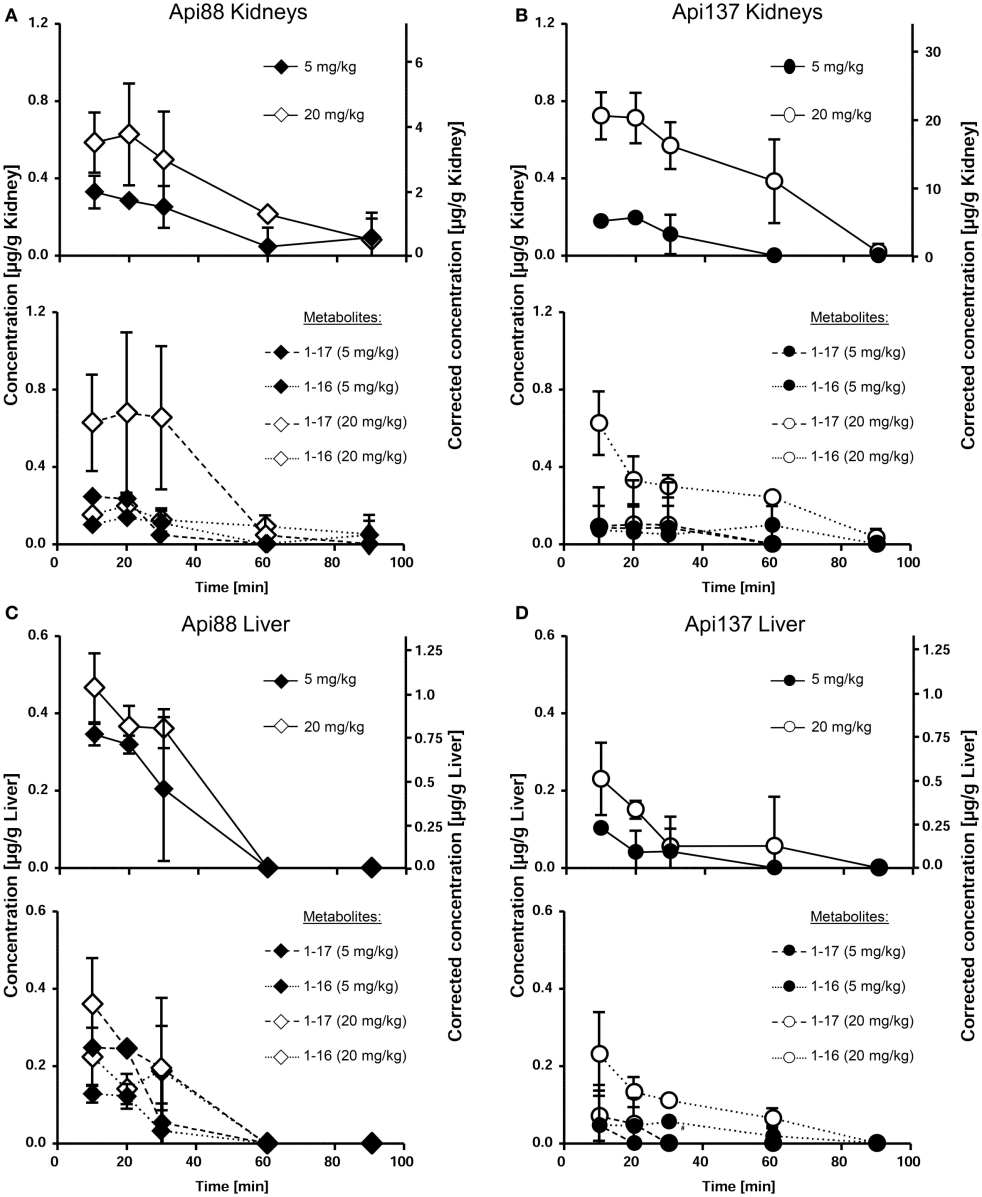
**Concentration profiles of Api88 (A,C)** and Api137 **(B,D)** and the corresponding metabolites Api1-17 and Api1-16 in kidney **(A,B)** and liver **(C,D)** homogenates. Api88 and Api137 were administered intraperitoneal (5 mg/kg and 20 mg/kg) and quantified in homogenates prepared from organs (*n* = 5) isolated 10, 20, 30, 60, and 90 min after administration. The hash (#) indicates that the Api1-17 value of one mouse was removed as outlier.

The low plasma levels and the corresponding high volumes of distribution (V_D_) of Api88 and Api137 partially attributed to C-terminal degradation resulted in recovery rates below 1% in blood relative to the injected peptide amounts (Tables S10, S11). The strong accumulation in kidneys and renal excretion was expected for the short polar peptides, as already noted for oncocin derivatives (Holfeld et al., 2015; Schmidt et al., 2016). Therefore, the tissue distribution was further investigated using homogenates of kidney, liver, and brain besides the appearance in urine. Api88 and Api137 were detected in kidney homogenates of the high dose group with almost stable peptide concentrations between 0.5 to 0.8 μg/g over the first 30 min post-injection (Figures 4A,B). The peptide concentrations decreased afterwards, but remained above the LOQ in some animals after 90 min. Four-fold lower doses reduced the kidney levels of Api137 around four-fold (0.18 to 0.11 μg/g), but only two-fold for Api88 (0.33 to 0.05 μg/g kidney). In contrast, the peptide levels in liver homogenates were dose-independent for both peptides and two-fold higher for Api88 (0.2–0.4 μg/g) compared to Api137 (0.1–0.2 μg/g) for the first three time points (Figures 4C,D). Metabolite Api1-16 was detected at slightly higher concentrations than Api1-17 in kidney and liver homogenates, but both were present at much lower levels than Api137 (Figures 4A–D). In contrast, Api88 and its metabolite Api1-17 were present in both homogenates at similar levels, while Api1-16 was lower concentrated (Figures 4A,C), which correlates to their plasma levels. Brain homogenates contained neither Api88 nor Api137, i.e., less than the LODs of 43 and 18 ng/mL homogenate, respectively, corresponding to ~89 and ~37 μg/g brain nor the two metabolites.

**Figure 5:**
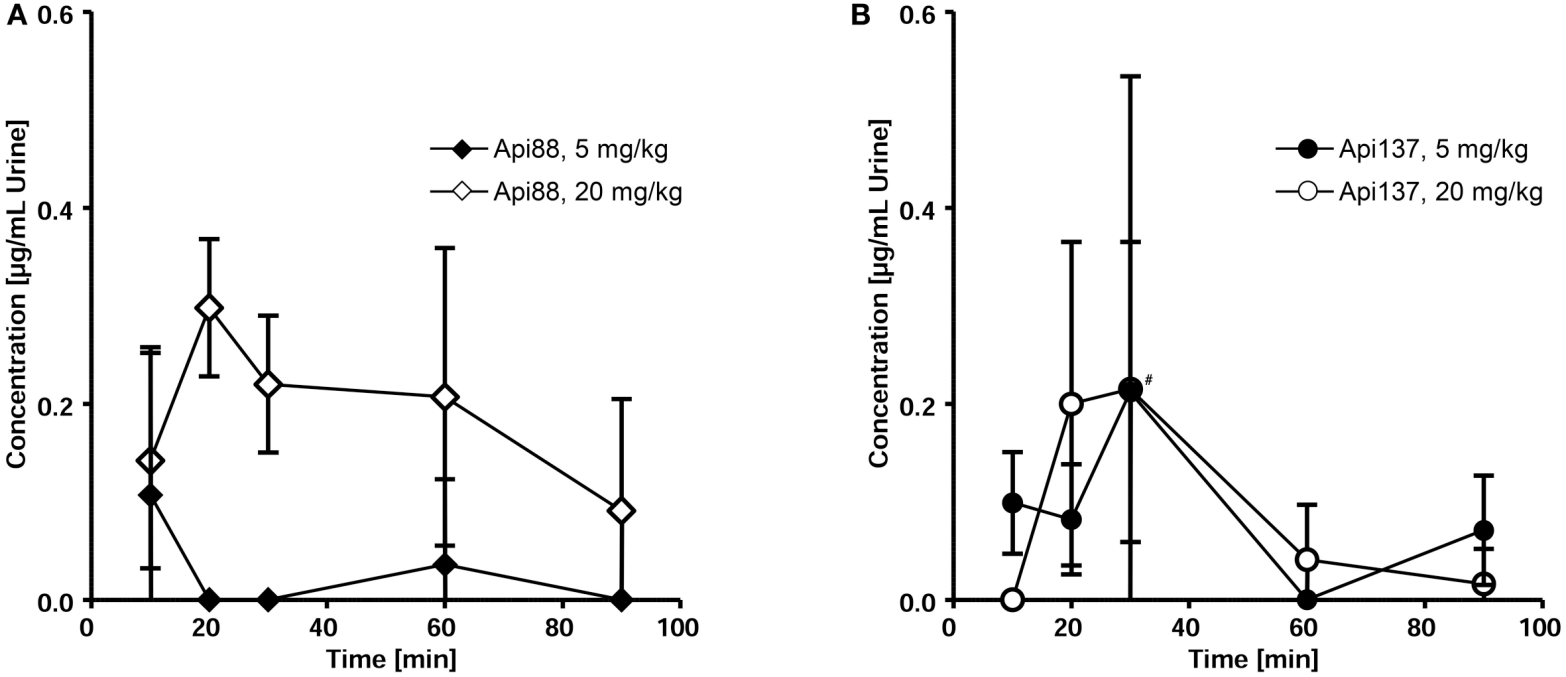
**Pharmacokinetics of Api88 (A)** and Api137 **(B)** in urine after intraperitoneal administration at doses of 5 and 20 mg/kg. Both peptides were quantified in urine (*n* = 3–7) spontaneously released after anesthetization. Concentrations of metabolites were below LOQ. The hash (#) indicates that one mouse was removed at this time point from the low dose group as outlier.

Three to seven mice of the studied groups spontaneously released urine during euthanasia. Api88 was present in all urine samples of the high-dose group at similar levels of ~0.2 μg/mL (Figure 5A). Urine collected in the low-dose group contained Api88 at the same level at the first time point, but it decreased afterwards to concentrations below LOD. The Api137 levels were similar for both dose groups showing peak concentrations of ~0.2 μg/mL in urine at 20 and 30 min, which decreased afterwards to levels below 0.1 μg/mL (Figure 5B). Metabolite Api1-17 was detected in the high-dose groups at intensities between the LOD and the LOQ and undetectable in the low dose group. The levels of Api1-16 were around the LOQ after 30 and 60 min in the low dose group, but similar to the concentrations of the full length peptides in the high dose groups.

### Peptide recovery in blood and organ homogenates

**Figure 6:**
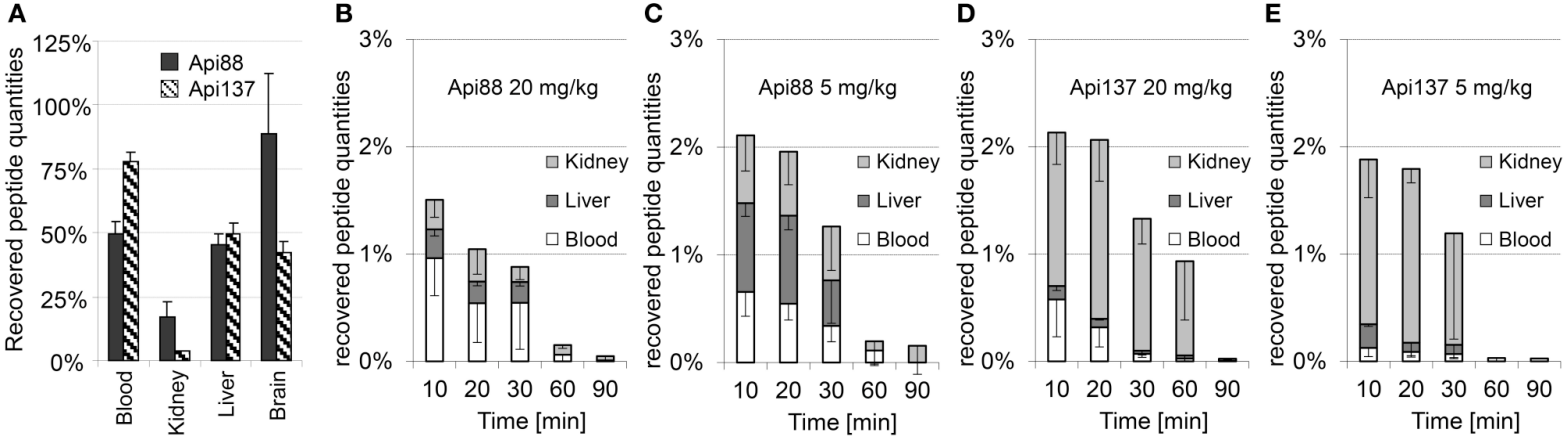
**Mean recovery rates (***n*** = 3) of Api88 and Api137 spiked to heparin blood or organ homogenates (A)** and mean percentage rates recovered in blood, liver, and kidneys of mice treated intraperitoneal with doses of 20 mg/kg **(B)** and 5 mg/kg of Api88 **(C)** or 20 mg/kg **(D)** and 5 mg/kg of Api137 **(E)**. Peptide distributions were calculated for plasma (one third of the whole mouse blood volume assumed to be 2.25 mL) and organs assuming that the peptides are present only in plasma and equally distributed in organ homogenates. Absolute quantities were calculated by assuming a total plasma volume of 25 mL/kg mouse weight and that organ volume in mL corresponds to the measured organ weight in g.

Peptide recoveries were determined by spiking untreated blood and homogenization buffer with either Api88 or Api137 prior to heparin plasma preparation or homogenization of organs obtained from untreated mice. Recovery rates for plasma were 49 ± 5 and 78 ± 5% for Api88 and Api137, respectively (Figure 6A, Table S12), which was similar to the recovery rates determined for liver (Api88: 45 ± 5%, Api137: 49 ± 4%) and brain homogenates (Api88: 89 ± 24%, Api137: 42 ± 4%). In contrast, the recovery rates were much lower in kidney homogenates (17 ± 6 and 3.5 ± 0.4%, respectively), which was previously noted for oncocin peptides as well (Schmidt et al., 2016). Metabolites identified by nanoRP-UPLC-ESI-Orbitrap-MS in the kidney homogenates were detected with small signal intensities relative to the full-length peptides indicating small peptide quantities (Table S13) that do not explain the low peptide recoveries in the kidneys. The low recoveries might be related to (*i*) further degradation to small peptides missed by the analytics, (*ii*) different metabolic pathways (e.g., oxidation), or (*iii*) strong binding to insoluble cell compartments that were removed by centrifugation after cell lysis (analysis of cell debris indicated only small peptide quantities, data not shown).

The recovery rates obtained from the spiked samples, were used to calculate the total amounts of intact peptides in blood, liver, and kidneys after intraperitoneal administration (Figures 6B–E, Tables S10, S11). The relative recovery in the studied organs and blood was only around 1.5 ± 0.6–2.1 ± 0.7% of the injected peptide amounts. Considering the mean weight of 29 g per mouse in this study and the weights of blood, liver, two kidneys, and brain with 2.2, 1.5, 0.5, and 0.5 g, respectively (~16% of the total weight), around 12% of the injected peptide quantities were detected in the samples collected at the first time point considering a homogeneous distribution in the whole animal. Notably, the recovery rates of Api88 and Api137 were similarly low and significantly lower than reported for oncocin analogs Onc72 and Onc112 administered intraperitoneal at a dose of 5 mg/kg (47 and 32%, respectively) in an equally performed pharmacokinetic study (Schmidt et al., 2016). In fact, C-terminal degradation of apidaecins could explain the difference, as metabolites of oncocins were detected at much lower amounts (Onc72) or not at all (Onc112). Nevertheless, the discrepancy between the bioavailability and peptide to metabolite ratios *in vivo* (especially for intraperitoneal administration) and the serum stability assay performed *in vitro* remained open.

### Peptide stability in peritoneal lavage

**Figure 7:**
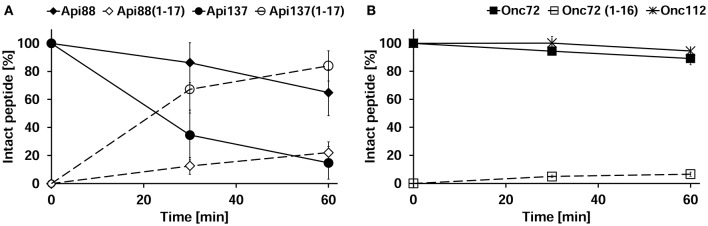
**Peritoneal lavage stability of apidaecins (A)** and oncocins **(B)**. Api88 (full diamonds), Api137 (full circles), Onc72 (full squares), and Onc112 (full asterisks) were incubated in lavage *ex vivo* and quantified after 30 and 60 min by RP-HPLC. Shown are the quantities of the incubated peptides (full symbols) and their main metabolites Api1-17 and Onc1-16 (open symbols) normalized to the initial peptide amounts determined at 0 min. The considered monoisotopic masses of the quadruply protonated metabolites were m/z 545.5 (Api1-17) and m/z 479.5 (Onc1-16) matching very well the signals at m/z 545.6 and 479.5, respectively, recorded with an Esquire HCT ESI-Iontrap-MS.

Peritoneal lavage was not collected during the pharmacokinetic study, as the main emphasis after euthanasia was the immediate collection of blood and perfused organs, whereas the discrepancy between *in vitro* degradation and *in vivo* metabolization was revealed only after completion of the animal studies. Thus, three untreated mice were euthanized and the peritoneum washed with PBS in order to obtain peritoneal lavage. Peptides Api88, Api137, Onc72, and Onc112 were incubated in aliquots of the lavage of each mouse *ex vivo* and analyzed for the added peptide and its degradation products after 0, 30, and 60 min. Surprisingly, Api137 was degraded very fast with a half-life time of ~20 min yielding Api1-17 as major metabolite (Figure 7A). Api88 was cleaved at the same position, but the amidated C-terminus reduced the degradation rate with more than 60% of Api88 still present after 1 h. The total amounts of intact peptide and Api1-17 reached in both cases almost 100% indicating that no other metabolites were produced in significant quantities. Onc72 and Onc112 were even more stable with more than 89% intact peptide present after 1 h (Figure 7B). Only for Onc72 the metabolite 1–14 was detected at higher quantities (7% relative to the intact peptide) after 60 min. It should be noted that the degradation was studied in lavage diluted with PBS at an unknown ratio and that the degradation rates in the peritoneum are most likely even higher. This nicely explains the unexpectedly low concentrations of Api137 in the pharmacokinetic study compared to Api88 and to oncocin peptides and similarly the surprisingly high concentrations of metabolites.

## Discussion

Apidaecin analogs Api88 and Api137 are similarly active against Gram-negative bacteria with a tendency of higher activity of Api88 against *K. pneumoniae* (Czihal et al., 2012; Berthold et al., 2013). However, Api88 carries a C-terminal amide that is responsible for its very fast proteolytic cleavage in serum, the supernatant obtained after complete coagulation and centrifugation of blood. The *in vitro* serum stability assay, which is an important *in vitro* criterion to select therapeutic peptides for systemic applications, yielded very short half-life times of 5 min for Api88, which indicate that the peptide is not well-suited for *in vivo* applications, while the 6 h for Api137 are very favorable (Berthold et al., 2013; Czihal et al., 2012). Taking into account the comparable minimal inhibitory concentration of ~4 μg/mL against *E. coli* ATCC 25922, it was predicted that Api137 exhibits a much better *in vivo* efficacy than Api88 in previous studies, because Api88 would be degraded rapidly after injection. Surprisingly, the *in vivo* efficacy of Api137 was only two-fold better with doses of 0.6 mg/kg Api137 rescuing all mice compared to 1.25 mg/kg Api88. Oncocin analogs Onc72 and Onc112, which possess MIC values of 16 and 4 μg/mL, respectively, against *E. coli* ATCC 25922 (Knappe et al., 2016c), rescued in the same infection model all animals at doses of 5 and 2.5 mg/kg, respectively (Schmidt et al., 2016). While the MIC values correlate well to the *in vivo* efficacies, the pharmacokinetics differed completely from our expectations considering the serum stability assays. Maximum plasma levels of Onc72 and Onc112 were up to 10-fold higher than that of Api88 and Api137 after intraperitoneal administration. For the high dose groups, the concentrations of Api1-16, Api1-17, and the intact peptide corresponded in total to around 10 and 16 μg/mL, which matches well Onc72 (18 μg/mL). The lower dose groups showed total maximum plasma levels (intact peptides plus metabolites) of 1.7 and 2.8 μg/mL for Api88 and Api137, respectively, which was around three-fold lower than reported for Onc72 (6 μg/mL) and Onc112 (8 μg/mL). Metabolites Api1-17 and Api1-16 are virtually inactive *in vitro* with MIC values of 64 μg/mL and >128 μg/mL, respectively. This is most likely related to the reduced bacterial uptake, as both metabolites bind equally well to the 70S ribosome and DnaK as the intact peptide (Berthold and Hoffmann, 2014; Krizsan et al., 2014). Although speculative, the bacterial uptake of the truncated sequences might change *in vivo*, as bacteria might activate different transporter systems under less favorable growing conditions. Additionally, host peptides increasing the bacterial membrane permeability might assist shortened peptides to enter bacteria. All other pharmacokinetic values depicting biodistribution in liver, kidneys, brain, and urine were lower values for both apidaecin analogs indicating a reduced overall recovery compared to both oncocin analogs. Although, the serum half-life times of Onc112 exceeded 8 h *in vitro*, Onc72 (3 h) was only half as stable (Knappe et al., 2011a) as Api137 (6 h), which contradicts the faster *in vivo* metabolization of Api137. The apparent discrepancy was solved by a peritoneal lavage *ex vivo* stability assay that revealed a fast degradation of Api137 indicating its fast metabolization in the peritoneum. Contrastingly, the concentration of Api137 was also slightly lower after intravenous application than for Api88. As expected from the lower serum stability of Api88, its metabolites were more abundant in blood, whereas no metabolites were detected for Api137. As renal excretion of both peptides appeared to be similar, distribution of Api88 and Api137 might be inconsistent as indicated by their different volumes of distribution.

Considering the pharmacokinetic data, the high *in vivo* efficacies of Api88 and Api137 are surprising. Thus, a drawback of intraperitoneal sepsis models commonly applied in antibiotic research has to be discussed, as infection and treatment used the same route and direct bacterial killing in the peritoneum starting 1 h post-infection appears very likely. Nevertheless, the model is accepted for evaluation of antimicrobials and the bacteria were well-distributed in blood and even organs prior to treatment (Frimodt-Møller et al., 1999; Knappe et al., 2012). Additionally, if the *in vitro* activity would translate to the activity in the peritoneum and sterilization of the peritoneum would be the only *in vivo* effect, Onc112 should show a much higher *in vivo* efficacy than Api137 due to its higher peritoneal stability. However, the plasma concentrations do not substantiate efficient treatment with either of the four PrAMPs when considering their *in vitro* antimicrobial activities determined with standard MIC-testing methods, which are most likely not the proper conditions to predict *in vivo* efficacies of PrAMPs. Synergistic effects with intrinsic antimicrobial substances produced by the host, e.g., AMPs like CRAMP, and immunomodulatory effects could also explain this discrepancy (Ostorhazi et al., 2013; Otvos and Ostorhazi, 2015; Knappe et al., 2016b). The latter effect was studied for Onc72, but no immunomodulatory effects were observed on unstimulated and lipopolysaccharide (LPS)-stimulated murine dendritic cells or murine macrophages (Fritsche et al., 2012). Only human macrophages and monocytes showed a reduction of LPS-induced TNFα release after treatment with Api88 and Api137 indicating a mild anti-inflammatory effect (Tavano et al., 2011; Keitel et al., 2013). Unfortunately, further data on immunomodulatory effects of Api137 on murine cells are missing.

Although speculative, the accumulation of the PrAMPs in the kidneys and possibly spleen (not tested) may lead to high local concentrations sufficient to kill bacteria in both organs that are responsible for clearance of bacteria in the blood stream. In this respect, the lower recovery rates of Api137 spiked to the buffer before kidney homogenization may indicate a strong binding to the organ structures, i.e., brush boarder membrane. Therefore, the recovered peptide amounts in kidneys of Api137 treated animals (2.4 μg/g) were slightly higher than in Api88 treated animals (1.8 μg/g). Notably, PrAMPs usually do not penetrate mammalian cells, but immune cells and HeLa cancer cells can internalize apidaecins and Bac7(1–35) after long incubation times (Tavano et al., 2011; Hansen et al., 2012; Pelillo et al., 2014; Bluhm et al., 2016). However, it is unlikely that PrAMPs internalized in kidney cells within 10 min (first time point of the pharmacokinetic study). More likely they bind to membrane structures or accumulate in the interstitial fluid of the kidney. Estimating the volume of both kidneys as 0.5 mL, the detected peptide amount would translate to kidney concentrations of 4.8 μg/mL, which is most likely higher in the interstitial volume and will easily kill bacteria surrounding kidney cells. In this respect, it has to be noted that the animals were carefully perfused with saline after euthanasia in order to remove blood from the organs and to measure only peptide amounts that are localized in the organs and not in blood. Considering all data presented above, it appears reasonable to assume that perfusion did also wash out peptides bound to the tissue or dissolved in the interstitial fluid. Therefore, the peptide amounts in the kidney with circulating blood might have been significantly higher.

Metabolization of therapeutic peptides in the peritoneum could be suppressed by formulating the peptides with EDTA or other biocompatible protease inhibitors providing maybe better *in vivo* efficacies and better pharmacokinetics. Alternatively, intramuscular and subcutaneous injections are valid alternative routes with depot effects and were already successfully applied to treat animals (Ostorhazi et al., 2010; Knappe et al., 2015). Even though, the unexpectedly good pharmacokinetic profile of Api88 can be only partially explained by its higher peritoneal stability. Further studies have to explain why Api88 appears much more stable in intravascular blood than in serum.

Besides the partially unexpected effects, the pharmacokinetic data may indicate that their therapeutic effects are related to their maximal blood concentrations and not to the time period above a certain threshold or the AUC. Our most recent data indicate an irreversible uptake of Api137 by bacteria into the cytosol (unpublished data), which may provide an explanation for their high *in vivo* efficacy, i.e., the peptide concentration could fall below the MIC as soon as a certain amount of PrAMP entered the cells. Earlier studies on fluorophore-labeled peptides indicated that Api88 enters *E. coli* cells faster than Api137 at high quantities, but the data might be influence by the N-terminally attached dye that reduced also MIC values (Czihal et al., 2012; Berthold and Hoffmann, 2014). Thus, further studies using RP-HPLC-ESI-MS techniques to study the cellular uptake of unmodified Api88, Api137, Onc72, and Onc112 and to determine the post-antibiotic effect of these peptides are necessary to quantify their *in vitro* activities in the context of the *in vivo* studies presented here.

## Conclusion

The data presented here together with literature data allowed comparing the *in vivo* efficacy of four PrAMPs, i.e., two apidaecin analogs Api88 and Api137 and two oncocin analogs Onc72 and Onc112 within and between both peptide families. The comparison relies on one infection model of peritoneal sepsis performed in one laboratory by the same persons revealing that the therapeutic efficacy increased in the following order: Api137 < Api88 < Onc112 < Onc72. This was not presumable from the order of stability determined *in vitro* in mouse serum (Onc112 > Api137 > Onc72 >> Api88) and the *in vitro* antimicrobial activities (Api88 > Api137 = Onc112 > Onc72). Both parameters are favoring Api137 and Onc112 well above Api88 and Onc72 with respect to their predicted *in vivo* efficacy. The stability in peritoneal lavage as third *in vitro* parameter ranked the peptides in a different order (Onc112 > Onc72 > Api88 > Api137) and explains partially the lower than expected performance of Api137 based on the initial *in vitro* data. There are a few possible explanations, e.g., organ accumulation, immunomodulatory effects, and irreversible bacterial uptake, to correlate the high *in vivo* efficacies with the relatively low plasma concentrations, which is commonly accepted as basis for antibiotic assessment. However, additional effects besides direct antimicrobial activity in the bloodstream have to be studied in more detail and the efficacy in different infection models will help to justify further clinical development of both apidaecin analogs and both oncocin analogs.

## Author contributions

RS: peptide synthesis, LC-MS method development, organ, and sample preparation, data evaluation, manuscript preparation. DK: peptide synthesis, infection model, stability assay in intraperitoneal lavage, data interpretation, design of the work, manuscript preparation. EW and EO: pharmacokinetics, sample preparation, proof reading manuscript. RH: Project supervision, design of the work, manuscript preparation.

### Conflict of interest statement

RH is a cofounder of AMP Therapeutics GmbH (Leipzig, Germany) and a member of their scientific advisory board. EW and DK were temporarily co-workers at AMP Therapeutics GmbH. Peptide sequences of Api137 and Api88 are patented under PCT/EP2008059512. The other authors declare that the research was conducted in the absence of any commercial or financial relationships that could be construed as a potential conflict of interest.
