# Supplementary material for: In vivo Efficacy and Pharmacokinetics of Optimized Apidaecin Analogs
Source: Front Chem. 2017 Mar 20;5:15. doi: 10.3389/fchem.2017.00015 (PMC5357639; doi:10.3389/fchem.2017.00015)
Supplement: Supplementary file 1 [file Presentation1.pdf]

Supplementary Material

Pharmacokinetics and In Vivo Efficacy of Optimized Apidaecin Derivatives

Rico Schmidt, Daniel Knappe, Eszter Ostorházi, and Ralf Hoffmann

Table of contents

Method M1: MRM transitions S2

Method M2: Sample preparation S2

Method M3: Recovery rates in plasma and organ homogenates S3

Method development and validation S4

Table S1. Sequences, monoisotopic masses, and MRM transitions of apidaecins S5

Table S2. Equipment for LC-MS analysis S6

Table S3. Ion source and MS analyzer settings for ESI-QqLIT-MS (4000 QTRAP®) S7

Table S4. MS/MS settings for ESI-QqLIT-MS (4000 QTRAP®) S8

Table S5. Ion source and MS analyzer settings for ESI-LTQ-Orbitrap XL™-MS S9

Table S6. LOD, LOQ, and LDR obtained from calibration without matrix S9

Table S7. Recovery and matrix effects after solid phase extraction S10

Table S8. Precision and accuracy of the RPC-MRM method S11

Table S9. LOD and LOQ obtained from calibration in urine and organ homogenates S12

Table S10. Recovery rates in blood and organ homogenates S13

Table S11. Api88 peptide recoveries calculated for blood, organs, and total animal S14

Table S12. In vivo metabolites in murine kidney homogenates S15

Table S13. Api137 peptide recoveries calculated for blood, organs, and total animal S16

Figure S1. ESI-QqLIT-MS and MS/MS of Api88 and Api137 S17

Figure S2. Influence of pH on signal intensities in MRM of Api88 and Api137 S18

Figure S3. Chromatograms of MRM-analysis of Api88 and Api137 S19

Figure S4. Pharmacokinetics of Api88/Api137 and their metabolites (5 mg/kg, i.p) S20

Figure S5. Exponential fittings of concentration profiles after i.p. administration. S21

Figure S6. Abundance of Api88/Api137 metabolites in blood after i.p. administration S22

Method M1: Multiple reaction monitoring (MRM) transitions for apidaecin peptides

Peptides (1 µg/mL) were dissolved in aqueous acetonitrile (25% or 60% v/v) containing either formate buffer (26 mmol/L, pH 2.6, 3.0, 3.3 or 3.9) or formic acid (0.1 % v/v) and infused (flow rate 5 µL/min, PHD 2600 syringe pump) into the ESI-QqLIT-MS (4000 QTRAP®) to optimize buffer composition and MRM. Transitions were selected from the tandem mass spectra (MS/MS) and confirmed on an ESI-QqTOF-MS (QSTAR® pulsar I) providing higher mass resolutions and mass accuracies than the QTRAP®. The settings for declustering potential (DP), collision potential (CE), and collision cell exit potential (CXP) were optimized by ramping the respective potentials using the compound optimization tool of the Analyst® software.

Method M2: Sample preparation

Frozen organs (5 per group) were homogenized in sterile PBS (500 µL) containing Protease Inhibitor Mix M (1% v/v; SERVA Electrophoresis GmbH, Heidelberg, Germany) using an Ultra-Turrax® T25 basic (IKA-Werke, Staufen, Germany, rotational speed 21,500 min^{-1}) for 1 min. Homogenates were sonicated on ice (35 % amplitude, mode: 5 s on; 5 s off for 1 min; Vibra-cell™ 75041, Thermo Fisher Scientific) to disrupt cells. After centrifugation (15 min, 21,460 x g, 4°C) the supernatants were stored at -80°C.

Aliquots of plasma (10 µL) or liver (10 µL), kidney (50 µL), and brain homogenates (100 µL) were spiked with isotope-labeled Api88, Api137, Api1-16, and Api1-17 (2.5 µg/mL). The samples were diluted fourfold with aqueous phosphoric acid (3.4 % v/v), loaded on an Oasis HLB 96-well solid phase extraction (SPE) plate (5 mg sorbent, Waters), washed with aqueous methanol (0.3 mL, 5% v/v), eluted with methanol (0.3 mL), and dried in a vacuum centrifuge (30 °C; SpeedVac, Eppendorf). The samples were dissolved prior analysis in aqueous acetonitrile (3% v/v) containing formic acid (0.1 % v/v).

Stock solutions (1 g/L) of the peptides and their corresponding isotope-labelled internal standards were prepared and diluted in water. Calibration and validation mixtures (10 µL) of unlabeled (0.025, 0.1, 0.5 or 2.5 µg/mL) and isotope-labeled peptides (2.5 µg/mL) were mixed with plasma or organ homogenates (10 to 100 µL) obtained from untreated animals for calibration.

Limits of detection (LOD) and quantification (LOQ) corresponded to the lowest peptide concentrations in water, plasma, or homogenates of brain, liver, and kidney providing a peak height exceeding the noise in the retention time window at least threefold (LOD) or ten times (LOQ). Recovery rates of SPE and matrix effects were determined by the peak areas of spiked

plasma samples and organ homogenates relative to equally concentrated peptide solutions in pure water. Concentrations of samples below the LOQ but still detectable were set to the value of the LOD, whereas signals with peak heights below the LOD were set to 0 µg/mL.

Method M3: Recovery rates in plasma and organ homogenates

Li-heparin blood samples obtained from untreated NMRI mice were spiked with Api88 or Api137 (final concentrations: 2.5 or 10 µg/mL), incubated (10 min, RT), and centrifuged (2,000x g, 3 min, RT) to obtain plasma. Frozen organs were homogenized with PBS (0.5 mL) containing protease inhibitor mix M (SERVA; 1 % v/v) and either Api88 (brain: 500 µL, 0.2 or 1 µg/mL, liver: 500 µL, 2 or 5 µg/mL, kidneys: 500 µL, 20 or 60 µg/mL) or Api137 (brain: 500 µL, 0.2 or 2 µg/mL, liver: 500 µL, 1 or 2.5 µg/mL, kidneys: 500 µL, 20 or 60 µg/mL). The analysis followed the protocol described above for pharmacokinetics samples. Recoveries were calculated by assuming a total blood (plasma) volume of 75 (25) mL per kg mouse weight [3] and that the measured organ weight (in g) corresponds to the organ volume (in mL).

Method development and validation

Apidaecin peptides were selectively and sensitively quantified based on the methods established for oncocins Onc72 and Onc112 (Schmidt R. et al JAC 2016). MRM based on triply charged precursor ions (Figure S1A and C) and a neutral loss of two dimethyl amine molecules cleaved from the N-terminal guanidine group as most intense fragment ions (Figure S1B and D). To avoid misinterpretation of possible cross talks, an additional MRM transition with an *m/z* above the *m/z* selected in quadrupole 1 was used (Table S4). MS settings were optimized in order to obtain the highest sensitivities (Table S4) and the pH of the eluents was elevated to 3.0 to increase the intensity of the signals corresponding to the triply protonated precursor ions (Figure S2). Interfering sample components, such as proteins, salts, and lipids, were removed by solid phase extraction (OASIS HLB plates) before the samples were analyzed by RP-HPLC-ESI-QqLIT-MS (flow rate: 0.2 mL/min, column temperature: 55°C, linear gradient from 2.7 to 20.7% acetonitrile containing 26 mmol/L formate buffer (pH 3.0) in five minutes) with an overall duration of 20 min. Polypropylene tubes were used for all handling steps, as the highly basic peptides adsorbed strongly to glass surfaces resulting in a hundred fold reduced sensitivity.

The method provided instrumental LODs of 14 and 28 ng/mL for Api88 and Api137, respectively, and only slightly higher LOQs (Table S6). The linear dynamic range spanned

more than two orders of magnitude up to 10 µg/mL. When all steps of the analytics were considered, the LODs increased for Api137 and even more for Api88 (Table S9), most likely through losses during SPE, which had recoveries between 35 and 56% for all four peptides, with higher recoveries for low concentrations (Table S7). The successful removal of isobaric components was confirmed by the absence of signals after injection of plasma (untreated animals) after SPE. The weak matrix effects on the signal intensities (<10%) with enhancing effects at low peptide concentrations further confirmed this. The precisions and accuracies met the requirements very well (Table S8) indicating that the developed method allowed a reliable and sensitive quantification of apidaecin peptides in biological samples. However, the LODs and LOQs increased in organ homogenates, especially for liver, and in urine samples (Table S9).

Table S1. Sequences, monoisotopic masses, transitions of MRM quantification, and analytical parameters of all apidaecin peptides analyzed in murine plasma samples.

**Peptide***	**Sequence#**	**[M+H]$^{+}$**		**MRM mass ranges**	
		**Theor.**	**Exp.**	**Q1**	**Q3**
Api88	gu-ONNRPVYIPRPRPPHPRL-NH$_2$	2290.36	2290.42	764.13<br>[M+3H]$^{3+}$	734.09<br>[MH$_3$-2 NHMe$_2$]$^{3+}$
Api88 IS	gu-ONNRPVYIPRPRPPHPRL-NH$_2$	2296.36	2296.47	766.13<br>[M+3H]$^{3+}$	736.09<br>[MH$_3$-2 NHMe$_2$]$^{3+}$
Api137	gu-ONNRPVYIPRPRPPHPRL-OH	2291.35	2291.41	764.45<br>[M+3H]$^{3+}$	734.41<br>[MH$_3$-2 NHMe$_2$]$^{3+}$
Api137 IS	gu-ONNRPVYIPRPRPPHPRL-OH	2297.35	2296.49	766.45<br>[M+3H]$^{3+}$	736.41<br>[MH$_3$-2 NHMe$_2$]$^{3+}$
Metabolite<br>1-17	gu-ONNRPVYIPRPRPPHPR-OH	2178.26	2178.30	726.80<br>[M+3H]$^{3+}$	696.70<br>[MH$_3$-2 NHMe$_2$]$^{3+}$
Metabolite<br>1-17 IS	gu-ONNRPVYIPRPRPPHPR-OH	2184.26	2184.31	728.80<br>[M+3H]$^{3+}$	698.70<br>[MH$_3$-2 NHMe$_2$]$^{3+}$
Metabolite<br>1-16	gu-ONNRPVYIPRPRPPHP-OH	2022.16	2022.24	674.70<br>[M+3H]$^{3+}$	644.70<br>[MH$_3$-2 NHMe$_2$]$^{3+}$
Metabolite<br>1-16 IS	gu-ONNRPVYIPRPRPPHP-OH	2028.16	2028.22	676.70<br>[M+3H]$^{3+}$	646.70<br>[MH$_3$-2 NHMe$_2$]$^{3+}$

* IS denotes isotope-labeled peptides

# gu denotes *N*,*N*,*N’*,*N’*-tetramethylguanidine, [$^{13}C_5$,^{15}N]-L-proline residues are underlined

Table S2. Equipment used for RP-HPLC-ESI-MS.

Company	Instrument/software
ABSciex Germany GmbH, Darmstadt, Germany	Analyst® 1.6 software
	ESI-QqLIT-MS (4000 QTRAP®)
	ESI-QqTOF-MS (QSTAR® pulsar I)
Harvard Apparatus INC. , Holliston, MA, USA	PHD 2600 syringe pump
Phenomenex® Ltd. Torrance, USA	Jupiter C_{18}-column (1 mm internal diameter, 150 mm length, 5 µm particle size, 30 nm pore size)
Thermo Fisher Scientific GmbH, Dreieich, Germany	ESI-LTQ-Orbitrap XL™-MS
	Xcalibur™ software
Waters GmbH, Eschborn, Germany	nanoACQUITY UPLC®
	BEH C_{18} nanoACQUITY UPLC® column (75 µm internal diameter, 100 mm length, 1.7 µm particle size, 13 nm pore size)
	MassLynx™ software
	Progenesis® QI software
	Alliance® 2695 HPLC system

Table S3. Turbo V^{TM} ion source and mass analyzer settings of the ESI-QqLIT-MS (4000 QTRAP®) for analyzing Api88, Api137, Api1-16, and Api1-17.

**Parameter**	**Turbo V^{TM} ion source settings**	
Sample introduction	Alliance® 2695 HPLC System	Syringe pump (PHD 2600)
Scan type	Multiple reaction monitoring	Q1- or product ion scan
Sample introduction flow rate	200 µL/min	5 µL/min
Ion spray voltage (IS)	5500 V	5500 V
Nebulizing gas (Gas 1)	50 psi	10 psi
Drying gas (Gas 2)	50 psi	-
Curtain gas (CUR)	30 psi	15 psi
Temperature (TEM)	650 °C	-
Interface heater	on	on
	**Mass analyzer settings**	
Quadrupole (Q1) resolution	Unit	Low (for product ion scan)
Quadrupole (Q3) resolution	Unit	-
Collision gas (CAD gas)	High	High (for product ion scan)

Table S4. MS/MS settings of the 4000 QTRAP® to quantify Api88, Api137, Api1-16, and Api1-17 as well as the corresponding isotope-labeled standard peptides.

**Peptide**	**Type**	**Precursor**		**Fragment**		**DP$_{opt}$ [V]**	**CE$_{opt}$ [V]**	**CXP$_{opt}$ [V]**
		**Ion**	***m/z***	**Ion**	***m/z***			
Api88	Quantifier	$[M+3H]^{3+}$	764.13	$[MH_3\text{-}2\ NHMe_2]^{3+}$	734.09	83	41	10
	Qualifier	$[M+3H]^{3+}$	764.13	$y^{2+}_{16} – NH_3$	1031.09	83	43	16
	IS	$[M+3H]^{3+}$	766.13	$[MH_3\text{-}2\ NHMe_2]^{3+}$	736.09	83	41	10
Api137	Quantifier	$[M+3H]^{3+}$	764.45	$[MH_3\text{-}2\ NHMe_2]^{3+}$	734.41	58	41	10
	Qualifier	$[M+3H]^{3+}$	764.45	y^{2+}_{16}	1040.10	58	39	16
	IS	$[M+3H]^{3+}$	766.45	$[MH_3\text{-}2\ NHMe_2]^{3+}$	736.41	58	41	10
Metabolite 1-17	Quantifier	$[M+3H]^{3+}$	726.80	$[MH_3\text{-}2\ NHMe_2]^{3+}$	696.70	58	37	8
	Qualifier	$[M+3H]^{3+}$	726.80	$[MH_3\text{-}NHMe_2]^{3+}$	711.70	58	35	10
	IS	$[M+3H]^{3+}$	728.80	$[MH_3\text{-}2\ NHMe_2]^{3+}$	698.70	58	37	8
Metabolite 1-16	Quantifier	$[M+3H]^{3+}$	674.70	$[MH_3\text{-}2\ NHMe_2]^{3+}$	644.70	58	35	8
	Qualifier	$[M+3H]^{3+}$	674.70	$y^{3+}_{16} – NH_3$	598.30	58	39	4
	IS	$[M+3H]^{3+}$	676.70	$[MH_3\text{-}2\ NHMe_2]^{3+}$	646.70	58	35	8

DP, CE, and CXP denote declustering, collision, and collision cell exit potentials, respectively.

Table S5. Ion source and mass analyzer settings applied on the ESI-LTQ-Orbitrap XL^{TM}-MS to identify metabolites of Api88 and Api137 in plasma samples.

Parameter	Setting
Ionization mode	Positive
Resolution	60000
Ion spray voltage (IS)	1500 V
Aux gas flow rate	1 arb
Capillary temperature	200 °C
Tube lens voltage	120 V
Mass-to-charge ratio (*m/z*) range	200 – 2000

Table S6. Analytical parameters calculated from a serial dilutions series of Api88 and Api137 analyzed by the optimized MRM method. Peptide solutions (10 µL) were prepared in water without any biological matrix.

Peptide	LOD	LOQ	LDR	R^2
Api88	14 ng/mL	15 ng/mL	$7*10^2$	0.9911
Api137	28 ng/mL	29 ng/mL	$3*10^2$	0.9988

LOD, LOQ, and LDR denote limit of detection, limit of quantification, and linear dynamic range, respectively.

Table S7. Recovery rates and matrix effects after SPE preparation of peptides spiked in murine heparin plasma and analyzed with the optimized MRM method.

Peptide	Concentration [ng/mL]	Recovery after SPE [%]	Matrix effects [%]
**Api88**	100	56 ± 16	13 ± 3
	600	50 ± 15	-1.9 ± 0.3
	5000	47 ± 11	-4.7 ± 0.5
**Api137**	100	45 ± 2	3.3 ± 0.2
	600	42 ± 4	-8.1 ± 0.4
	5000	41 ± 6	-5.2 ± 0.2
**Api1-17**	100	53 ± 6	3.2 ± 0.4
	600	49 ± 6	-4.5 ± 0.1
	5000	46 ± 8	-0.64 ± 0.02
**Api1-16**	100	42 ± 2	3.6 ± 0.3
	600	36 ± 4	-4.4 ± 0.2
	5000	35 ± 5	1.9 ± 0.1

Table S8. Precision and accuracy of the optimized MRM method applied to murine heparin plasma samples calculated from three triplicates repeated on three consecutive days.

Peptide	Concentration [ng/mL]	Precision		Accuracy [%]
		Intraday	Interday	
Api88	50	9 – 10%	9%	104 ± 6
	250	1 – 2%	1%	94 ± 1
	1000	1 – 2%	1%	99 ± 2
	5000	1 – 2%	1%	100 ± 1
Api137	50	8 – 11%	10%	116 ± 17
	250	4 – 7%	3%	89 ± 6
	1000	1 – 6%	2%	98 ± 1
	5000	1 – 7%	3%	100 ± 1
Api1-17	50	5 – 18%	8%	98 ± 13
	250	3 – 6%	1%	97 ± 5
	1000	1 – 2%	2%	100 ± 3
	5000	1 – 6%	3%	100 ± 1
Api1-16	50	12 – 14%	9%	94 ± 11
	250	2 – 4%	3%	94 ± 3
	1000	1 – 4%	1%	101 ± 1
	5000	1 – 5%	3%	101 ± 1

Table S9. LODs and LOQs determined for Api88, Api137, and their two main metabolites in urine and organ homogenates. The sample volumes of urine and liver, kidney, and brain homogenates were 10, 10, 25, and 50 µL, respectively.

**Peptide**	**LODs / LOQs [ng/mL]**				
	Plasma	Liver	Kidney	Brain	Urine
Api88	55 / 59<br>(74 / 78)#	216 / 246	87 / 101	43 /56	197 / 213
Api137	35 / 39	191 / 266	76 / 93	18 / 34	69 / 114
Api1-17	14 / 19	50 / 85	45 / 100	6 / 33	27 / 58
Api1-16	16 / 26	77 / 232	33 / 42	13 / 18	35 / 107

Studies using intravenous and intraperitoneal administrations were performed at different times and thus slightly different LODs/LOQs determined for each analysis period were applied.

Table S10. Peptide amounts recovered in mice samples after intraperitoneal administration of Api88. Quantities were corrected by the recovery rates determined for plasma and each organ homogenate (Table S12). Absolute quantities were calculated by assuming a total plasma volume of 25 mL/kg mouse weight and organ volumes determined in mL corresponded to the measured organ weights in g. Percentages of mean injected peptide amounts recovered in respective samples are provided in brackets.

Dose	Time [min]	Mean mouse weight [g]	Mean injected peptide amount [µg]	Peptide amount [µg]				
				Blood	Liver	Kidney	Urine	Total
5 mg/kg	10	27.8	138.9	0.9 (0.7%)	1.1 (0.8%)	0.9 (0.6%)	0 – 0.009 (0 – 0.006%)	**2.9 (2.1%)**
	20	28.3	141.6	0.8 (0.5%)	1.2 (0.8%)	0.8 (0.6%)	0 (0%)	**2.8 (2.0%)**
	30	27.3	136.5	0.5 (0.3%)	0.6 (0.4%)	0.7 (0.5)	0 (0%)	**1.7 (1.3%)**
	60	26.3	131.6	0.1 (0.1%)	0 (0%)	0.1 (0.1%)	0 – 0.006 (0 – 0.005%)	**0.3 (0.2%)**
	90	27.4	137.0	0 (0%)	0 (0%)	0.2 (0.2%)	0 (0%)	**0.2 (0.2%)**
20 mg/kg	10	32.3	646.9	6.2 (1.0%)	1.7 (0.3%)	1.8 (0.3%)	0 – 0.02 (0 – 0.003%)	**9.7 (1.5%)**
	20	30.6	612.9	3.3 (0.5%)	1.2 (0.2%)	1.9 (0.3%)	0.0004 – 0.02 (0.0001 – 0.003%)	**6.4 (1.0%)**
	30	29.4	588.9	3.2 (0.5%)	1.2 (0.2%)	0.8 (0.1%)	0.008 – 0.013 (0.001 – 0.002%)	**5.3 (0.9%)**
	60	31.8	636.6	0.4 (0.1%)	0 (0%)	0.6 (0.1%)	0 – 0.03 (0 – 0.005%)	**1.0 (0.2%)**
	90	30.3	606.9	0.1 (<0.1%)	0 (0%)	0.2 (<0.1%)	0 – 0.05 (0 – 0.009%)	**0.3 (<0.1%)**

Table S11. Peptide amounts recovered in mice samples after intraperitoneal administration of Api137. Quantities were corrected by the recovery rates determined for plasma and each organ homogenate (Table S12). Absolute quantities were calculated by assuming a total plasma volume of 25 mL/kg mouse weight and organ volumes determined in mL corresponded to the measured organ weights in g. Percentages of mean injected peptide amounts recovered in respective samples are provided in brackets.

**Dose**	**Time [min]**	**Mean mouse weight [g]**	**Mean injected peptide amount [µg]**	**Peptide amount [µg]**				
				**Blood**	**Liver**	**Kidney**	**Urine**	**Total**
**5 mg/kg**	10	31.5	157.4	0.2 (0.1%)	0.3 (0.2%)	2.4 (1.5%)	0 – 0.005 (0 – 0.003%)	**3.0 (1.9%)**
	20	31.2	155.9	0.1 (0.1%)	0.1 (0.1%)	2.5 (1.6%)	0 – 0.01 (0 – 0.008%)	**2.8 (1.8%)**
	30	27.2	136.1	0.1 (0.1%)	0.1 (0.1%)	1.4 (1.0%)	0.004 – 0.04 (0.003 – 0.03%)	**1.6 (1.2%)**
	60	27.5	137.6	<0.1 (<0.1%)	0 (0%)	0 (0%)	0 (0%)	**<0.1 (<0.1%)**
	90	28.1	140.6	<0.1 (<0.1%)	0 (0%)	0 (0%)	0 – 0.02 (0 – 0.01%)	**<0.1 (<0.1%)**
**20 mg/kg**	10	30.1	601.4	3.5 (0.6%)	0.8 (0.1%)	8.6 (1.4%)	0 (0%)	**12.8 (2.1%)**
	20	30.4	607.4	1.9 (0.3%)	0.5 (0.1%)	10.1 (1.7%)	0 – 0.004 (0 – 0.001%)	**12.5 (2.1%)**
	30	32.1	641.7	0.5 (0.1%)	0.2 (<0.1%)	7.9 (1.2%)	0 – 0.004 (0 – 0.001%)	**8.5 (1.3%)**
	60	29.6	592.3	0.2 (<0.1%)	0.2 (<0.1%)	5.2 (0.9%)	0 – 0.006 (0 – 0.001%)	**5.5 (0.9%)**
	90	28.5	569.1	<0.1 (<0.1%)	0 (0%)	0.1 (<0.1%)	0 – 0.006 (0 – 0.001%)	**0.1 (<0.1%)**

Table S12. Recovery rates of Api88 and Api137 determined in plasma and organ homogenates (n = 3) collected from untreated NMRI mice. Solutions of Api88 (blood: 10 µL, 25 and 100 µg/mL, brain: 500 µL, 0.2 or 1 µg/mL, liver: 500 µL, 2 or 5 µg/mL, kidneys: 500 µL, 20 or 60 µg/mL) and Api137 (blood: 10 µL, 2.5 and 10 µg/mL, brain: 500 µL, 0.2 or 2 µg/mL, liver: 500 µL, 1 or 2.5 µg/mL, kidneys: 500 µL, 20 or 60 µg/mL) were added prior to homogenization and centrifugation. Peptide concentrations were calculated based on the assumption that peptides were equally distributed in plasma (one third of the 2.5 mL blood volume of mice) and present only in the supernatant of the homogenates, i.e. neglecting peptide contents remaining in the pellets. The total peptide amount in mice was calculated from the recovery rates shown in bold.

**Sample**	**Api88**		**Api137**	
	High concentration	Low concentration	High concentration	Low concentration
Plasma	(48 ± 6)%	**(49 ± 5)%**	(77 ± 1)%	**(78 ± 4)%**
Kidney	(14.0 ± 0.2)%	**(16.7 ± 6)%**	(2.7 ± 0.7)%	**(3.5 ± 0.4)%**
Liver	(34 ± 3)%	(45 ± 5)%	(47 ± 4)%	**(49 ± 4)%**
**Brain**	**0%**	**(89 ± 24)%**	**0%**	**(42 ± 4)%**

Table S13. Metabolites of Api88 and Api137 determined in homogenates prepared from kidneys collected 10 min, 30 min, and 60 min after intraperitoneal peptide administration. Kidney samples obtained from three mice per time point were pooled and analyzed by nanoRP-UPLC-ESI-LTQ-Orbitrap XL^{TM}-MS. Api88 and Api137 were not detected in any sample.

Metabolite	Charge state	m/z	Retention time [min]	sequence[c]
1-9	2	592.85	19.1	guan-ONNRPVYIP-OH
1-11	3	479.95	12.0	guan-ONNRPVYIPRP-OH
1-13	4	423.51	13.2	guan-ONNRPVYIPRPRP-OH
1-14	4	447.77	13.9	guan-ONNRPVYIPRPRPP-OH
1-16	4/5	506.30/405.24	14.0	guan-ONNRPVYIPRPRPPHP-OH
2-16	4	453.26	13.9	H-NNRPVYIPRPRPPHP-OH
7-16	3	410.57	12.6	H-YIPRPRPPHP-OH
8-14	2	416.76	12.0	H-IPRPRPP-OH
8-18	3	445.60[a]/445.94[b]	13.6[a]/13.5[b]	H-IPRPRPPHPRL-NH_2/OH
9-18	3	407.92[a]	16.7[a]	H-PRPRPPHPRL-NH_2/OH
12-18	2	436.27[a]/436.76[b]	11.1[a]/10.8[b]	H-RPPHPRL-NH_2/OH

[a] Retention time and *m/z* obtained for a C-terminal metabolite of Api88
[b] Retention time and *m/z* obtained for a C-terminal metabolite of Api137
[c] C-terminal sequences were always amidated and free acids when derived from Api88 and Api137, respectively.

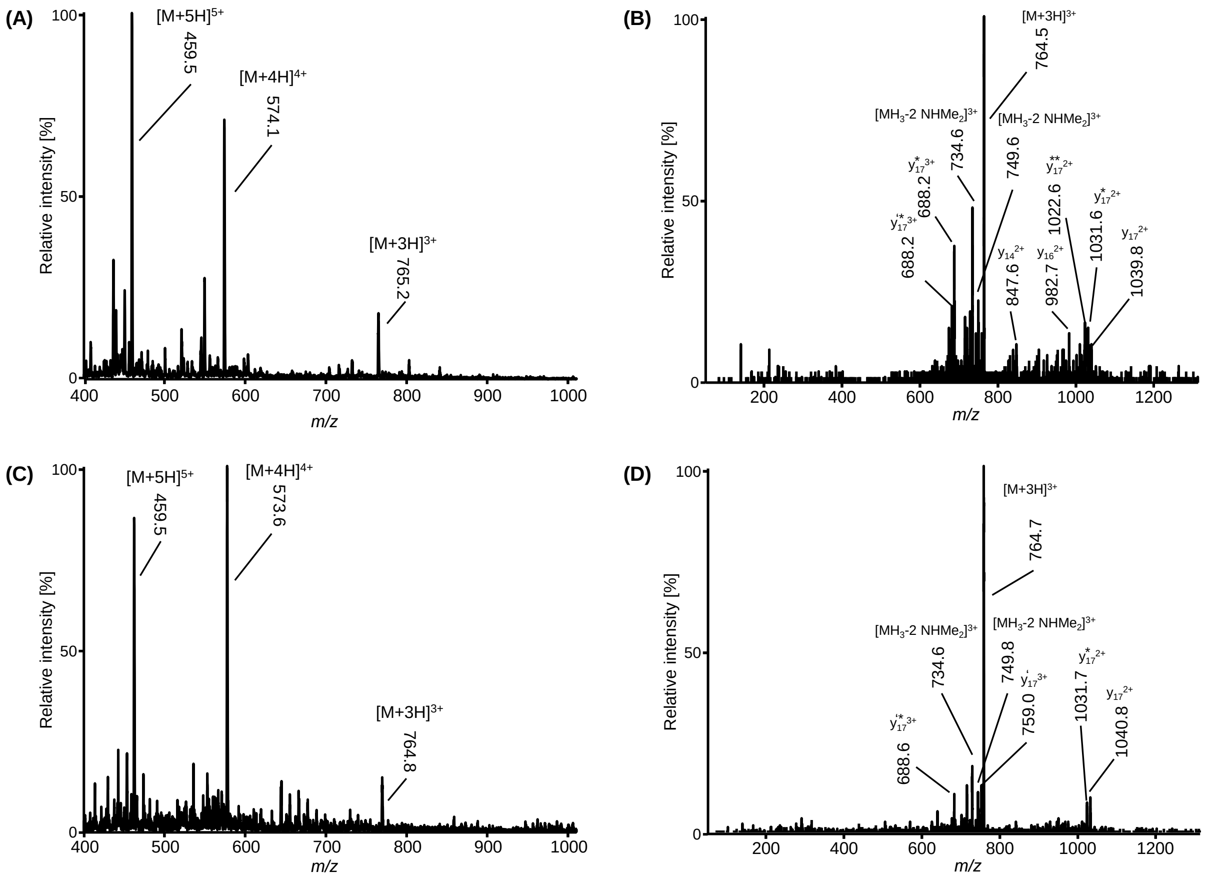

Figure S1. ESI-QqLIT-MS (A and C) and -MS/MS (B and D; $[M+3H^+]^{3+}$) of Api88 (A and B) and Api137 (C and D). Api88 and 137 were infused with a syringe pump (5 µL/min). Fragmentation relied on collision potential setting of 45 V (Api88) and 40 V (Api137) using nitrogen as collision gas. Mass shifts indicating neutral losses of ammonia and water are indicated by asterisks and apostrophes, respectively.

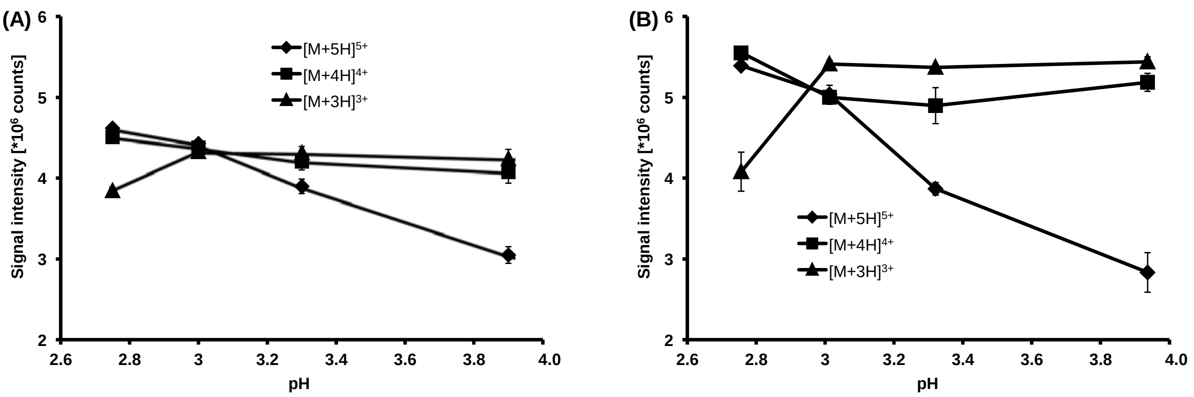

Figure S2. Influence of pH on signal intensities in MRM. Mean signal intensities (n = 3) of different precursor charge states were determined for Api88 (A) and Api137 (B) for a pH range from 2.6 to 4.0 in the presence of formate/formic acid. Peptides were dissolved in 25% (v/v) aq. acetonitrile containing 26 mmol/L formate buffer, infused (5 µL/min), and analyzed in Q1 scan mode on a 4000 QTRAP®.

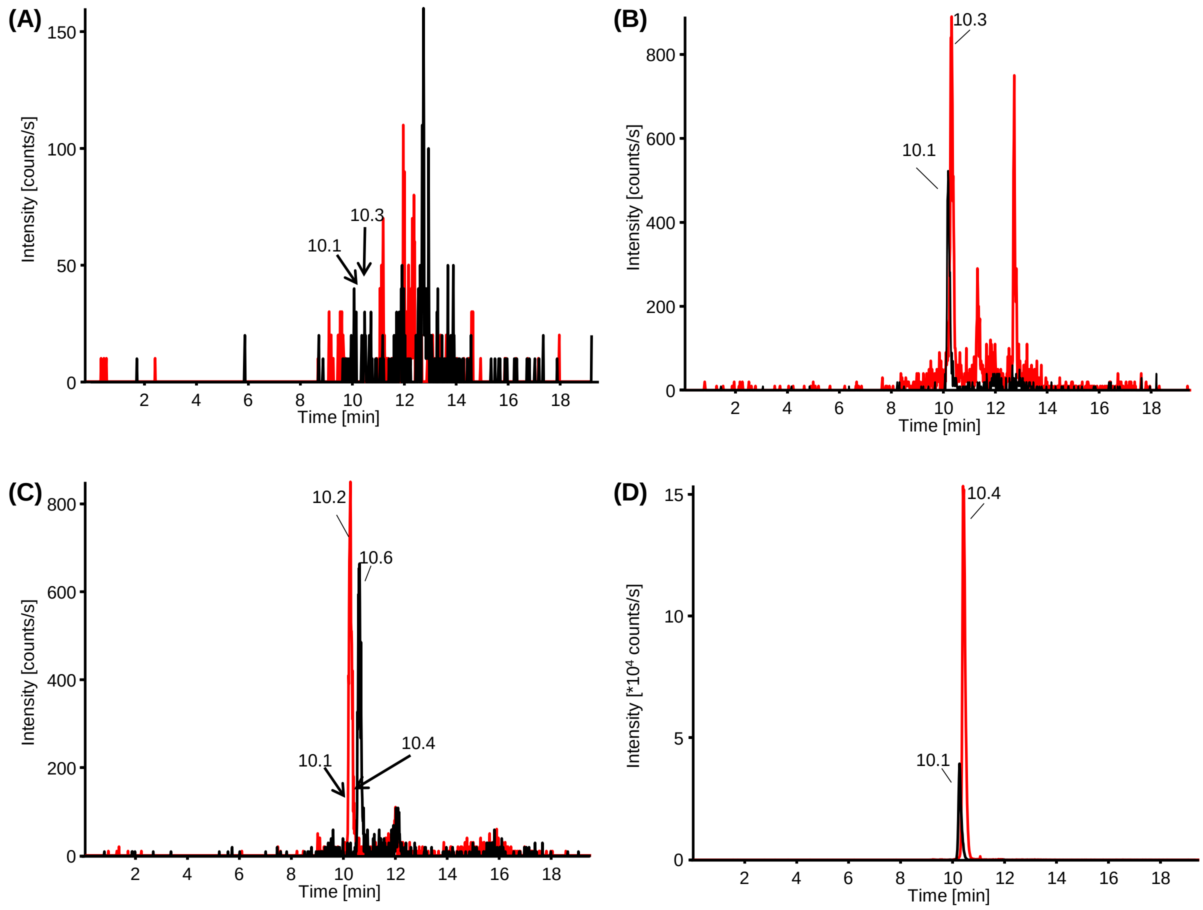

Figure S3. Chromatograms of Api88 (black) and Api137 (red) (A and B) and their isotope-labeled internal standards (C and D, respectively) recorded by the optimized MRM. Plasma extracts (10 µL) were analyzed without (A and C) and after addition of native (0.1 µg/mL, B) or isotope-labelled Api88 and Api137 (2.5 µg/mL, D).

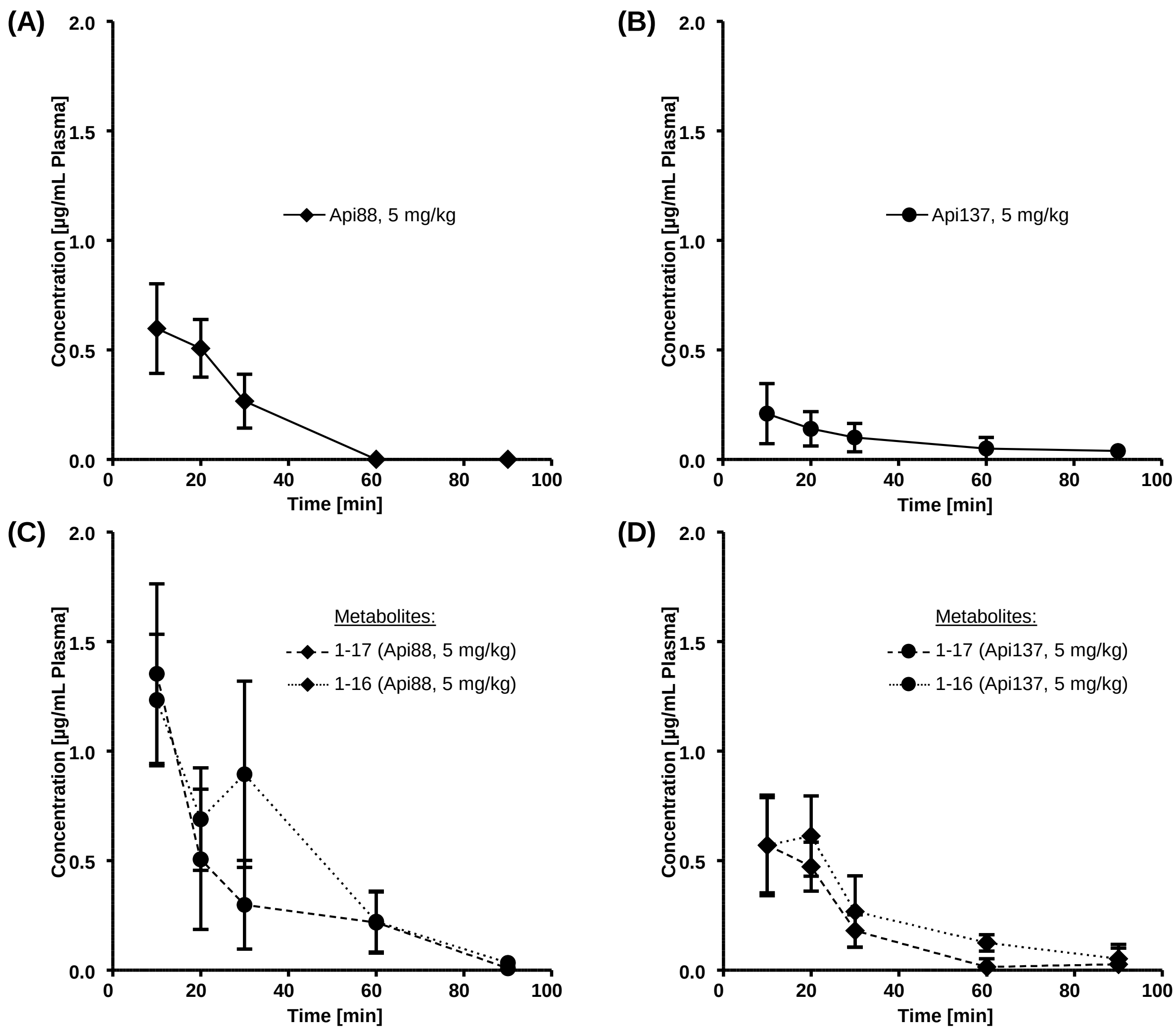

Figure S4. Pharmacokinetics of Api88 (A), Api137 (B), and metabolites 1-17 and 1-16 (C and D) after intraperitoneal administration at doses of 5 mg/kg BW. Peptides were quantified by RPC-ESI-QqLIT-MS/MS in plasma samples collected at 10, 20, 30, 60, and 90 min post injection (n = 7).

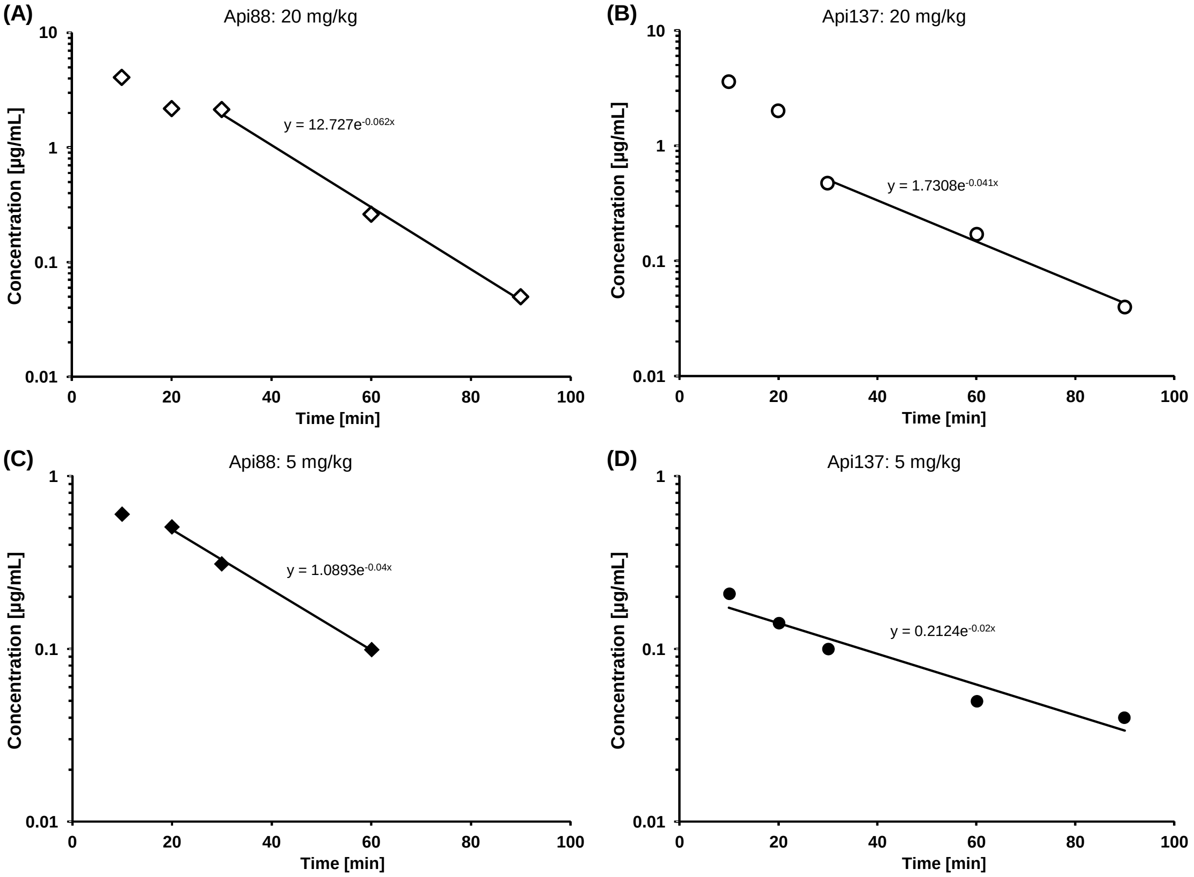

Figure S5: Pharmacokinetics of Api88 (A and C) and Api137 (B and D) intraperitoneally administered at doses of 20 mg/kg BW (A and B) and 5 mg/kg BW (C and D). Graphs and formula of exponential fittings used to determine the pharmacokinetic parameters were calculated with PKSolver. The exponential elimination time range was automatically set by the software. Peptides were quantified by RPC-ESI-QqLIT-MS/MS in plasma samples collected at 10, 20, 30, 60, and 90 min post injection (n = 7).

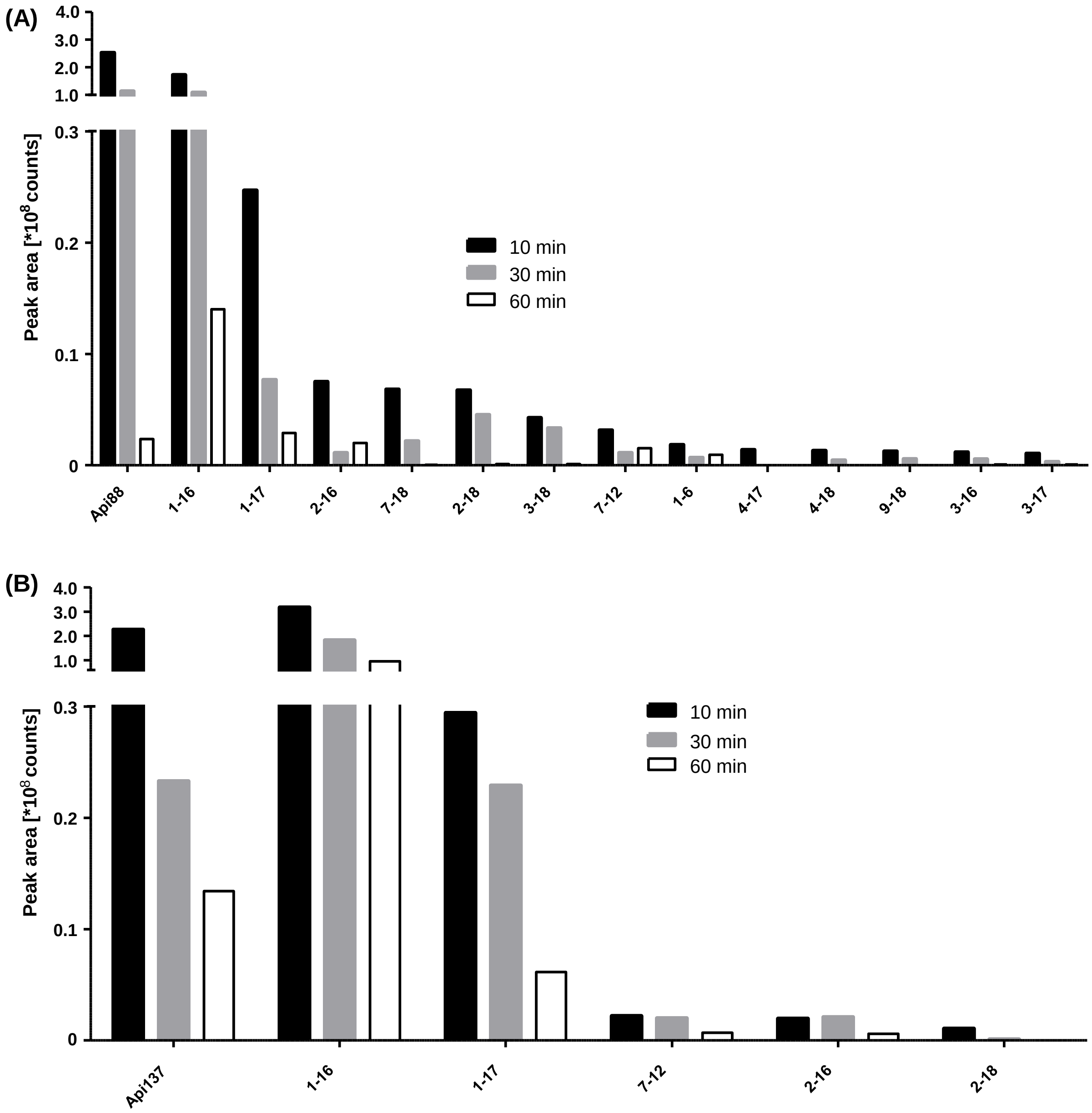

Figure S6. Peak areas of Api88 (A), Api137 (B), and the respective metabolites were determined in heparin plasma samples using the extracted ion chromatograms (XICs, *m/z* ± 0.01). Blood samples were collected 10, 30, and 60 min after intraperitoneal administration of Api88 or Api137 (three mice per time point), pooled, and analyzed by nanoRP-UPLC-LTQ-Orbitrap XL^{TM}-MS. Peak areas represent the sum of all peak areas of a charge series of a given peptide or metabolite normalized to the total peak areas. Shown are metabolites with peak areas larger than 10^6 counts.
